# cGAS Inhibits ALDH2 to Suppress Lipid Droplet Function and Regulate MASLD Progression

**DOI:** 10.1002/advs.202508576

**Published:** 2025-10-03

**Authors:** Ying Wang, Yu Deng, Jianfeng Chen, Quentin Hahn, David S. Umbaugh, Zhigang Zhang, Yanqiong Zhang, Sarah E. Rowe, Lupeng Li, Laura E. Herring, Brian P. Conlon, Edward A. Miao, Blossom Damania, Anna Mae Diehl, Pengda Liu

**Affiliations:** ^1^ Lineberger Comprehensive Cancer Center and Curriculum in Genetic and Molecular Biology The University of North Carolina at Chapel Hill Chapel Hill NC 27599 USA; ^2^ Department of Integrative Immunobiology Duke University Durham NC 27710 USA; ^3^ Lineberger Comprehensive Cancer Center and Department of Biochemistry and Biophysics The University of North Carolina at Chapel Hill Chapel Hill NC 27599 USA; ^4^ Department of Biochemistry and Biophysics The University of North Carolina at Chapel Hill Chapel Hill NC 27599 USA; ^5^ Department of Medicine Duke University Durham NC 27710 USA; ^6^ Lineberger Comprehensive Cancer Center Department of Microbiology and Immunology and Center for AIDS Research The University of North Carolina at Chapel Hill Chapel Hill NC 27599 USA; ^7^ Department of Microbiology and Immunology The University of North Carolina at Chapel Hill Chapel Hill NC 27599 USA; ^8^ UNC Proteomics Core Facility Department of Pharmacology University of North Carolina at Chapel Hill Chapel Hill NC 27599 USA; ^9^ Department of Integrative Immunobiology Department of Molecular Genetics and Microbiology Department of Pathology and Department of Cell Biology Duke University School of Medicine Durham NC 27710 USA

**Keywords:** ALDH2, cGAS, HFD, lipid droplets, MASLD

## Abstract

Cyclic GMP‐AMP synthase (cGAS) is a cytosolic DNA sensor essential for host defense against microbial infections, but its role beyond innate immunity remains unclear. Here, a non‐canonical function of cGAS in regulating aldehyde metabolism and lipid homeostasis is identified. This is demonstrated that cGAS directly binds to and suppresses ALDH2 (aldehyde dehydrogenase 2), a key enzyme in ethanol metabolism and lipid peroxidation. Loss of cGAS activates ALDH2, thereby enhancing ethanol tolerance in mice. Elevated ALDH2 activity upon cGAS loss increases aldehyde conversion into acetyl‐CoA, promoting histone acetylation and transcription of lipid synthesis genes, which drives lipid droplet accumulation in cells and in cGas^‐/‐^ mouse livers. These lipid droplets confer resistance to ferroptosis but simultaneously induce ER stress, impairing STING (stimulator of interferon genes) activation. Functionally, cGas^‐/‐^ mice fed with a modified high‐fat diet develop exacerbated metabolic dysfunction‐associated steatotic liver disease (MASLD), characterized by excessive lipid droplet accumulation in livers compared to wild‐type controls. In human MASLD patient cohorts, increased cGAS but reduced ALDH2 mRNA expression is observed relative to healthy individuals. Together, this findings uncover a previously unrecognized role of cGAS in metabolic regulation, independent of its innate immune function. By suppressing ALDH2, cGAS controls lipid droplet biogenesis and stress responses, with direct implications for MASLD pathogenesis.

## Graphical abstract

cGAS is a cytosolic DNA sensor known for innate immune defense. cGAS binds and suppresses ALDH2 are showed, limiting acetyl‐CoA production. cGAS loss activates ALDH2, promoting histone acetylation, lipogenesis, and lipid droplets accumulation. cGas^‐/‐^ mice on a high‐fat diet show signs of worsened MASLD. This reveals a novel immunity‐independent role of cGAS linking metabolism and epigenetic regulation.

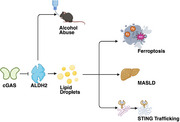

## 1 Introduction

cGAS is a major cytosolic dsDNA sensor^[^ ^1^, ^2^ ^]^ that detects aberrantly presented cytosolic DNA, originating from both foreign pathogens including viruses^[^ ^1^ ^]^ and bacteria,^[^ ^3^ ^]^ as well as endogenous sources such as damaged mitochondria^[^ ^4^ ^]^ or genomic DNA.^[^ ^5^ ^]^ Upon binding dsDNA, cGAS is activated to produce the secondary messenger 2′3′‐cGAMP,^[^ ^6^ ^]^ which subsequently binds to the ER‐resident protein STING.^[^ ^7^ ^]^ This interaction facilitates STING trafficking to the Golgi and the recruitment of downstream signaling factors, ultimately driving production of type‐I interferons (IFNs), induction of interferon‐stimulated genes (ISGs)^[^ ^1^ ^]^ and secretion of proinflammatory cytokines. Together, these responses are critical for controlling infection and initiating adaptive immunity.^[^ ^8^, ^9^, ^10^ ^]^ Genetic studies have shown that *cGas^‐/‐^* or *Sting^gt/gt^* mice are highly susceptible to DNA virus infection due to impaired IFNs and ISG induction.^[^ ^2^ ^]^ In line with its essential role in innate immune activation, aberrant hyperactivation of cGAS is linked to autoimmune disorders such as Aicardi‐Goutières syndrome (AGS) and systemic lupus erythematosus (SLE), both marked by excessive IFN signaling.^[^ ^4^, ^11^ ^]^ Strikingly, deletion of *cGas* in *Trex1^‐/‐^* mice largely rescues AGS ;^[^ ^12^ ^]^ and SLE‐^[^ ^4^ ^]^ like phenotypes, underscoring a critical role of cGAS in autoimmune pathogenesis.

On the other hand, cGAS activation can be beneficial in cancer therapy by enhancing antitumor immunity. cGAS‐mediated innate immune signaling improves antigen presentation in dendritic cells (DCs),^[^ ^13^ ^]^ thereby increasing immune cell infiltration into tumors. Notably, 2′3′‐cGAMP has been shown to synergize with immune checkpoint blockade (ICB) therapy, enhancing its efficacy in reducing the growth of xenografted B16 melanoma tumors.^[^ ^14^ ^]^ Given its dual roles in both autoimmune diseases and tumor immunity, cGAS activity must be tightly regulated to maintain balance across diverse cellular contexts. This regulation occurs through multiple layers, including post‐translational modifications of cGAS,^[^ ^9^, ^15^ ^]^ interactions with cGAS‐binding proteins [9], and mechanisms controlling cGAS cellular localization.^[^ ^16^, ^17^ ^]^ Dysregulation of cGAS regulatory mechanisms has been implicated in various human disorders and represents a potential therapeutic opportunity.^[^ ^18^, ^19^ ^]^ To date, most known cGAS binding proteins are either viral proteins that inhibit cGAS activation to evade immune surveillance^[^ ^20^ ^]^ or host factors that modulate its activity. For example, G3BP1 (GTPase‐activating protein SH3 domain‐binding protein 1) enhances DNA binding and cGAS activation,^[^ ^21^ ^]^ whereas the autophagy protein Beclin‐1 binds to and restrains cGAS to prevent overactivation.^[^ ^22^ ^]^ In contrast, relatively few bacterial proteins have been reported to regulate cGAS. Previously, we identified streptavidin, a protein secreted by the soil bacterium *Streptomyces avidinii* as an enhancer for cGAS activation by promoting DNA binding.^[^ ^23^ ^]^ However, because *Streptomyces avidinii* is not a human pathogen, it remains unclear whether and how proteins from human pathogenic bacteria modulate cGAS activation during infection.

Beyond its role in pathogen defense, activation of cGAS/STING innate immune signaling has been implicated in diverse pathological processes. For example, it contributes to insulin resistance driven by mitochondrial DNA (mtDNA) released during DsbA‐L (disulfide‐bond A oxidoreductase‐like protein) deficiency,^[^ ^24^ ^]^ promotes aging and neurodegeneration through chronic inflammation,^[^ ^25^ ^]^ and induces steatosis and fibrosis in the livers of mice fed methionine/choline‐deficient or high‐fat diets (HFDs). Notably, this latter effect is uniquely mediated by STING activation in Kupffer cells.^[^ ^26^ ^]^ More recently, emerging roles of cGAS beyond innate immunity have begun to be recognized. For example, nuclear cGAS has been reported to suppress DNA damage repair by directly binding and inhibiting PARP1^[^ ^27^ ^]^ and to regulate genome stability by decelerating replication forks.^[^ ^28^ ^]^ Nonetheless, whether and how cGAS exerts additional innate immunity‐independent function in fatty liver disease remains unclear. Here, we report that cGAS binds to and suppresses ALDH2 activity. thereby limiting lipid droplet function and protecting mice from developing MASLD (non‐alcoholic fatty liver disease) induced by methionine/choline‐deficient high‐fat diets (HFDs). These findings highlight a previously unrecognized role of cGAS in metabolic regulation with potential therapeutic implications.

## 2 Results

### 2.1 S. Aureus ALDH is a cGAS‐Binding Protein that Facilitates cGAS Activation in Cells

To identify novel cGAS‐interacting proteins from human pathogenic bacteria, we focused on *Staphylococcus aureus* (*S. aureus*), a Gram‐positive bacterium that colonizes ≈30% of the human population,^[^ ^29^ ^]^ commonly residing in the nasal cavity, skin, and other tissues.^[^ ^30^, ^31^ ^]^ *S. aureus* is an opportunistic pathogen and a major cause of skin infections, pneumonia, and soft tissue infections, ranking among the leading contributors to global morbidity and mortality, particularly with the emergence of methicillin‐resistant *S. aureus* (MRSA).^[^ ^32^ ^]^ The absence of an approved vaccine^[^ ^33^ ^]^ further complicates the control of *S. aureus* infections, presenting significant clinical and research challenges. While prior studies have highlighted critical roles for c‐di‐AMP/STING/IFN‐β signaling in regulating *S. aureus* infection outcomes,^[^ ^34^ ^]^ the contribution of cGAS to host responses against *S. aureus* infections remains poorly defined.

**Figure 1:**
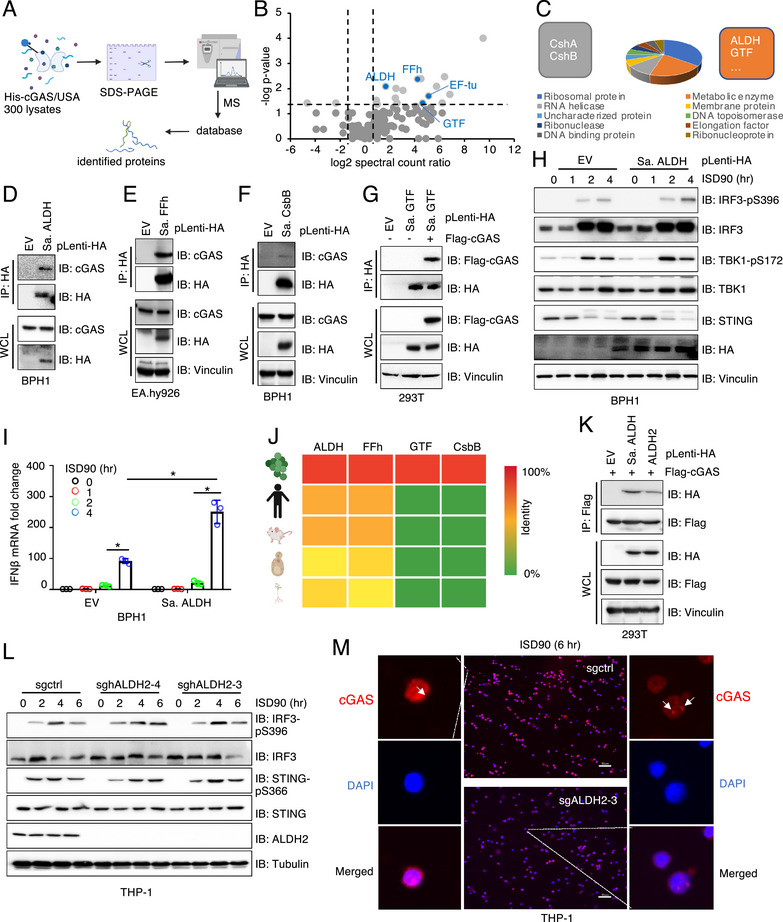
S. *aureus* ALDH promotes cGAS activation in cells, whereas human ALDH2 does not affect cGAS activation in response to dsDNA stimulation. A) A schematic representation of the experimental procedure for mass spectrometry analysis to identify cGAS binding bacterial proteins. See method for details. B) A volcano plot illustrating *S. aureus* proteins bound to cGAS, with top‐ranking candidates highlighted in red. C) A 3D pie chart summarizing the functional categories of *S. aureus* proteins bound to cGAS. D–G) Immunoblot (IB) analysis of HA immunoprecipitations (IPs) and whole cell lysates (WCL) from cells stably expressing (D) *S. aureus* ALDH, (E) *S. aureus* Ffh, (F) *S. aureus* CsbB, and (G) *S. aureus* GTF. H) IB analysis of WCL from BPH1 cells stably expressing either an empty vector or *S. aureus* ALDH, treated with 5 µg/ml ISD90 for the indicated time points. I) qPCR analysis of IFN‐β mRNA levels in BPH1 cells stably expressing either an empty vector or *S. aureus* ALDH, treated with ISD90 for the indicated time points. Error bars were calculated as mean+/‐SD, n=3 biological replicates. *p < 0.05 (one‐way ANOVA test). J) A schematic representation summarizing the categories of *S. aureus* proteins that bind cGAS. K) IB analysis of WCL from HEK293T cells transfected with the indicated DNA constructs, with cells collected 48 h post‐transfection. L) IB analysis of WCL from control or endogenous ALDH2‐depleted THP‐1 cells treated with ISD90 for the indicated time points. M) Representative confocal microscopy images of control or endogenous ALDH2‐depleted THP‐1 cells treated with ISD90 for 6 h, stained with a cGAS antibody. Scale bar = 50µm.

We incubated bacterially purified His‐tagged full‐length human cGAS proteins with *S. aureus* USA300 bacteria lysates in vitro and cGAS binding bacterial proteins were identified by mass spectrometry (**Figures** **1**A; S1A, Supporting Information). Using a cut‐off of ≈2‐fold enrichment in cGAS‐pulldown vs. control, we defined 21 candidate cGAS‐interacting proteins (Figure 1B). Functional annotation suggested that these bacterial proteins participate in diverse biological processes, including protein translation, RNA processing, metabolic regulation and others (Figure 1C). Among these candidates, we successfully validated several interactions in cells, including Sa. ALDH (aldehyde dehydrogenase, Figure 1D), Sa. FFh (signal recognition particle, Figure 1E), Sa. CsbB (lipoteichoic acid‐specific glycosylation protein, Figure 1F) and Sa. GTF (glycosyltransferase family 2 protein, Figure 1G). Notably, expression of Sa. ALDH in HEK293T (Figure S1B, Supporting Information), BPH1 (Figure 1H) or EA.hy926 cells (Figure S1C, Supporting Information) enhanced cGAS/STING activation, as evidenced by increased pIRF3 and/or pTBK1 levels, along with elevated IFN‐β transcription in BPH1 cells (Figure 1I). These results suggest that Sa. ALDH binds to and promotes activation of human cGAS in cells.

To investigate whether Sa. ALDH binding to host cGAS influences bacterial replication, infection, or host innate immune responses, we used a cutaneous infection mouse model, in which STING has been previously implicated.^[^ ^35^ ^]^ WT and *cGas^‐/‐^* mice were subcutaneously injected with 2x10^7^ USA300 MRSA, and tissues were collected from day 1 to day 3 post injection to determine CFU (colony‐forming unit) as bacterial burdens and IFN‐β secretion (Figure S1G, Supporting Information). Analysis of CFUs in skin, liver, spleen and kidney at day 1 to day 3 showed no significant differences between WT and *cGas^‐/‐^* mice (Figure S1H–K, Supporting Information). Similarly, IFN‐β production in these tissues were comparable between the two groups (Figure S1L–O, Supporting Information). Thus, in this cutaneous skin infection mouse model, cGAS does not appear to influence the host response to *S. aureus* infection, making it unsuitable for investigating the functional consequence of Sa. ALDH binding to host cGAS in vivo.

Interestingly, the *S. aureus* proteins that bind cGAS can be divided into two groups: those with high sequence conservation across species (eg. ALDH and FFh) and proteins unique to *S. aureus* (such as GTP and CsbB) (Figure 1J). Given that Sa. ALDH is evolutionarily conserved and shares a greater sequence similarity with ALDH2 compared to the other 18 mammalian ALDH proteins, we next investigated whether mammalian ALDH2 also binds to and regulates cGAS activation. Because the *S. aureus* ALDH/cGAS interaction can occur only during infection, with ALDH released into the host cytoplasm at an unknown stoichiometry, we will not pursue further investigation of this interaction but will instead focus on the physiological significance of the human ALDH2/cGAS interaction.

### 2.2 Mammalian ALDH2 is a cGAS‐Binding Protein but does not Regulate cGAS Activation

ALDH2 is a mitochondrial protein^[^ ^36^, ^37^ ^]^ primarily expressed in the liver^[^ ^38^ ^]^ but also present at significant levels in other organs.^[^ ^39^ ^]^ It plays a key role in ethanol metabolism by converting ethanol‐derived acetaldehyde into acetic acid while reducing NAD⁺ to NADH, predominantly in the liver.^[^ ^40^, ^41^, ^42^ ^]^ The resulting acetic acid is further converted into acetyl‐CoA, which participates in metabolic pathways and facilitates the acetylation of histone and non‐histone proteins.^[^ ^43^ ^]^ Beyond acetaldehyde detoxification, ALDH2 is crucial for oxidizing lipid peroxidation‐derived aldehydes, such as 4‐hydroxynonenal (4‐HNE)^[^ ^44^, ^45^ ^]^ and malondialdehyde (MDA),^[^ ^46^, ^47^ ^]^ into less toxic metabolites like 4‐hydroxy‐2‐nonenoic acid (4‐HNA) and malonic acid (MOA), respectively.

We found that similar to Sa. ALDH, human ALDH2 also bound cGAS in cells (Figure 1K). However, depletion of endogenous ALDH2 in THP‐1 cells did not significantly affect pIRF3 nor pSTING signals induced by ISD90 stimulation (Figure 1L), cGAS foci formation (Figure 1M; Figure S1P, Supporting Information), or IFN‐β production (Figure S1Q, Supporting Information). Likewise, depletion of endogenous ALDH2 in BPH1 cells (Figure S1R, Supporting Information) failed to affect cGAS/STING activation in response to ISD90 (Figure S1S,T, Supporting Information) or RNA stimulation by poly I:C (Figure S1U, Supporting Information). Notably, ALDH2 depletion had no impact on cGAS protein abundance (Figure S1D, Supporting Information). These data cumulatively suggest that although mammalian ALDH2 binds to cGAS, it does not regulate cGAS/STING activation upon DNA or RNA challenges.

### 2.3 cGAS Suppresses Mammalian ALDH2 Activation In Vitro and In Vivo

**Figure 2:**
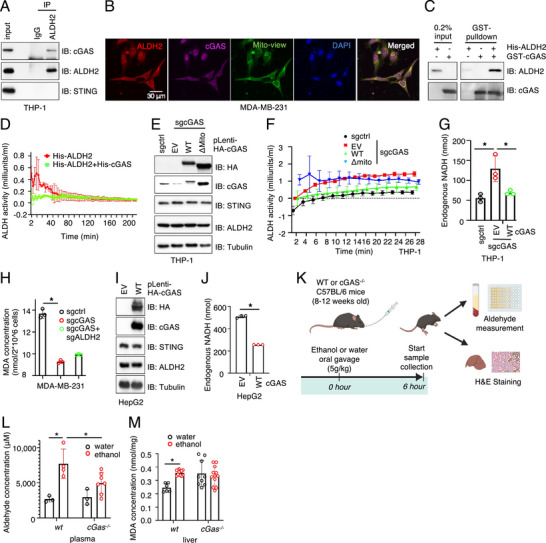
cGAS binds to ALDH2 to inhibit its activity A) IB analysis of WCL from THP‐1 cells immunoprecipitated using ALDH2 antibody. B) Representative confocal images of parental MDA‐MB‐231 cells stained with cGAS, ALDH2 antibodies, and Mitochondrial marker. Scale bar = 30µm.C) GST pull‐down assay showing that purified GST‐cGAS proteins bind to purified His‐ALDH2 in vitro. D) Enzymatic activity assay of purified His‐ALDH2 incubated with purified His‐cGAS proteins, further analyzing ALDH2 enzymatic activity. E) IB analysis of WCL from THP‐1 cells depleted of cGAS and re‐expressing either wild‐type cGAS (cGAS‐WT) or a mitochondria‐localization signal‐deleted cGAS mutant via lentiviral infection. Cells were selected with 2 mg ml^−1^ blasticidin for 72 h to eliminate non‐infected cells. F) Enzymatic activity of ALDH2 and G) endogenous NADH levels were measured in each cell line. Error bars were calculated as mean+/‐SD, n=3 biological replicates. *p < 0.05 (one‐way ANOVA test). H) MDA‐MB‐231 cells were depleted of cGAS, either re‐expressed with cGAS‐WT, or depleted of ALDH2, and MDA concentrations were measured in each cell line. Error bars were calculated as mean+/‐SD, n=3 biological replicates. *p < 0.05 (one‐way ANOVA test). I) IB analysis of WCL from HepG2 cells overexpressing either an empty vector or cGAS‐WT. J) Endogenous NADH levels were analyzed in cells from K). Error bars were calculated as mean+/‐SD, n=3 biological replicates. *p < 0.05 (one‐way ANOVA test). (K) A schematic representation of the mouse ethanol oral gavage experimental procedure: Refer to the method section for details. L) Serum aldehyde concentrations and M) liver MDA concentrations were measured. Error bars were calculated as mean+/‐SD, n=3 biological replicates. *p < 0.05 (one‐way ANOVA test).

Since ALDH2 does not regulate cGAS activation, we next investigated whether cGAS modulates ALDH2 activity. We confirmed that ALDH2 interacted with cGAS at endogenous levels in THP‐1 cells (**Figure** **2**A) and observed partial co‐localization of ALDH2 with cGAS on mitochondria in MDA‐MB‐231 cells by confocal imaging (Figure 2B). To further validate a direct interaction, we purified recombinant cGAS and ALDH2 proteins from bacteria (Figure S2A–C, Supporting Information) and found that full‐length cGAS proteins efficiently pulled down ALDH2 proteins in vitro (Figure 2C). Furthermore, full‐length human cGAS proteins inhibited ALDH2 enzymatic activity in vitro (Figure 2D). These data cumulatively suggest that cGAS binds to ALDH2 to suppress ALDH2 activation. We further depleted endogenous cGAS in THP1 (Figure 2E) or MDA‐MB‐231 cells (Figure S2D, Supporting Information) and found cGAS depletion caused elevated ALDH2 activities in cells (Figure 2F; Figure S2E, Supporting Information) without affecting ALDH2 protein abundance (Figure 2E; Figure S2D, Supporting Information). Reintroduction of WT‐cGAS largely reversed this effect, as evidenced by ALDH2 activity assays (Figure 2F) and monitoring of endogenous NADH levels (during ALDH2 catalysis of aldehydes into acetates, NAD^+^ is converted into NADH^[^ ^48^ ^]^), a readout of ALDH2 catalytic activity (Figure 2G). Interestingly, a reported mitochondria‐localization signal‐deleted cGAS mutant (aa161‐190 deletion^[^ ^49^ ^]^), termed as ΔMito‐cGAS, failed to suppress ALDH2 activity in cGAS depleted cells (Figure 2E,F; Figure S2F,G, Supporting Information), highlighting the importance of mitochondrial localization for cGAS‐mediated suppression. Additional depletion of ALDH2 partially rescued the MDA consumption resulting from cGAS depletion (due to elevated ALDH2 activity) (Figure 2H; Figure S2H, Supporting Information), confirming the specificity of these assays in evaluating ALDH2 activity. In human liver cancer cell lines HepG2 and PLC/PRF/5 that express little or no cGAS proteins, stably reconstitution of cGAS via lentiviral infection (Figure 2I) led to marked suppression of ALDH2 activity, as indicated by reduced NAD^+^ to NADH conversion (Figure 2J). Collectively, these results suggest that cGAS binds to and suppresses ALDH2 activity both in vitro and in cells.

Considering the well‐characterized ALDH2 function in metabolizing ethanol^[^ ^40^, ^41^, ^42^ ^]^ and detoxifying harmful aldehydes,^[^ ^44^, ^45^ ^]^ we next examined whether *cGas^‐/‐^* mice exhibit enhanced ALDH2 activity in vivo. *WT* and *cGas* ^‐/‐^ C57BL/6 mice were administered ethanol (5 g kg^−1^) via oral gavage and plasma and liver samples were collected 6 h later for analysis (Figure 2K). No notable changes in tissue morphology or neutrophil enrichment were observed (Figure S2I, Supporting Information). In *WT* mice, ethanol administration markedly increased aldehyde concentrations in plasma and MDA levels in the liver; these increases were absent in *cGas* ^‐/‐^ mice (Figure 2L,M). Because aldehyde and MDA levels are inversely correlated with ALDH2 activity, these findings suggest that *cGas* deletion enhances ALDH2 activity, promoting the conversion of aldehydes and MDA into non‐toxic products.

### 2.4 cGAS Binds and Prevents ALDH2 Tetramerization/Oligomerization to Suppress ALDH2 Activity

**Figure 3:**
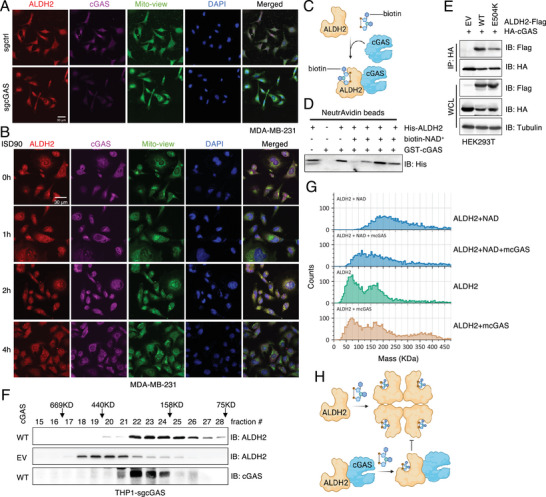
cGAS suppresses ALDH2 activity via inhibiting ALDH2 tetramer formation A) Representative confocal microscopy images of control and cGAS‐depleted MDA‐MB‐231 cells stained with the indicated antibodies. Scale bar = 30µm. B) Representative confocal microscopy images of parental MDA‐MB‐231 cells treated with ISD90 at the indicated time points and stained with the indicated antibodies. Scale bar = 30µm. C) A schematic illustration of the experiment setup. Refer to the method section for details. D) IB analysis of neutravidin beads pulldown products from reactions from (C). E) IB analysis of WCL and HA‐IPs derived from HEK293T cells transfected with the indicated DNA constructs. Cells were collected 48 h post‐transfection. F) IB analysis of indicated fractions from size exclusion chromatography analysis using either sgctrl or sgcGAS THP1 cell lysates. G) Representative mass photometry analysis of the indicated samples showing how cGAS interferes with ALDH2 tetramer formation. H) A cartoon illustration of our proposed model, where NAD+ binding facilitates ALDH2 tetramerization, while cGAS binding to ALDH2 prevents this process.

We next investigated the mechanism(s) by which cGAS binds to and inhibits ALDH2 activity. Compared with WT‐cGAS, cGAS mutants deficient in DNA binding (KKEA, K173E/R176E/K407E/K411A), enzymatic activity (GSAA, G212AS213A),^[^ ^23^ ^]^ or nucleosome binding (R255E),^[^ ^17^ ^]^ all showed comparable binding to ALDH2 in cells (Figure S3A–C, Supporting Information). These results suggest that neither the innate immunity nor nucleosome binding capacity is required for cGAS binding to ALDH2. Additional cGAS mutants, including K173A (deficient in K27‐linked cGAS ubiquitination and activation^[^ ^50^ ^]^), K285A (deficient in DNA binding site C^[^ ^51^ ^]^) or K414A (deficient in K414 acetylation that suppresses cGAS activation^[^ ^52^ ^]^) also bound ALDH2 similarly to WT‐cGAS in cells (Figure S3D, Supporting Information), supporting the notion that cGAS may bind ALDH2 independent of its established roles in innate immunity. Consistent with this notion, mitochondrial‐localized cGAS represented the primary population that binds ALDH2, as deletion of the mitochondrial localization signal in cGAS impaired its interaction with ALDH2 in cells (Figure S3E,F, Supporting Information), which echoes our previous observation that ΔMito‐cGAS failed to suppress ALDH2 activity (Figure 2F). Depletion of endogenous cGAS did not alter ALDH2 cellular localization (**Figure** **3**A), and DNA stimulation with ISD90, which triggered cGAS foci formation and activation, did not affect ALDH2 localization either (Figure 3B). Because ALDH2 binds NAD^+^ to cataly aldehyde metabolism,^[^ ^48^ ^]^ we next tested whether cGAS affects substrate loading. Biotin‐NAD^+^ efficiently pulled down recombinant His‐ALDH2 proteins in vitro, and the addition of GST‐cGAS proteins did not significantly impact NAD^+^ loading to ALDH2 (Figure 3C,D). These findings indicate that cGAS binding does not impact ALDH2 substrate loading.

ALDH2 is a mitochondrial enzyme^[^ ^36^, ^37^ ^]^ that functions as a tetramer composed of two dimers, with each dimer formed by two identical monomers.^[^ ^53^ ^]^ A common ALDH2 variant, ALDH2*2 (ALDH2‐E504K), was frequently observed in Asian population,^[^ ^54^ ^]^ is catalytic deficient due to impaired tetramer formation and enhanced protein turnover.^[^ ^55^ ^]^ Individuals carrying the ALDH2*2 allele often experience symptoms such as dizziness, headaches, and facial flushing even after moderate ethanol consumption,^[^ ^56^, ^57^ ^]^ which arise from the rapid accumulation of acetaldehyde in the liver that diffuses into the bloodstream. We found that, compared to WT‐ALDH2, the ALDH2‐E504K mutant (deficient in ALDH2 tetramerization) exhibited reduced binding to cGAS in cells (Figure 3E), suggesting that ALDH2 tetramerization contributes to cGAS recognition. In addition, in cGAS‐depleted THP‐1 cells, endogenous ALDH2 migrated into higher molecular weight protein complexes (Figure 3F), indicating cGAS may prevent the formation of higher order ALDH2 assemblies. Because NAD^+^ promotes formation of active ALDH tetramers,^[^ ^58^ ^]^ we further investigated whether cGAS interferes with NAD^+^‐driven ALDH2 tetramer formation. To this end, in vitro mass photometry analysis showed that recombinant mouse cGAS (mcGAS) proteins^[^ ^59^ ^]^ significantly blocked NAD^+^‐induced tetramerization of bacterially purified ALDH2 proteins (Figure 3G). Collectively, these findings suggest that cGAS binds to ALDH2 and inhibits its tetramerization, thereby suppressing its catalytic activity (Figure 3H).

### 2.5 cGAS Depletion Increases Lipid Droplets in Cells Depending on ALDH2 Activity

**Figure 4:**
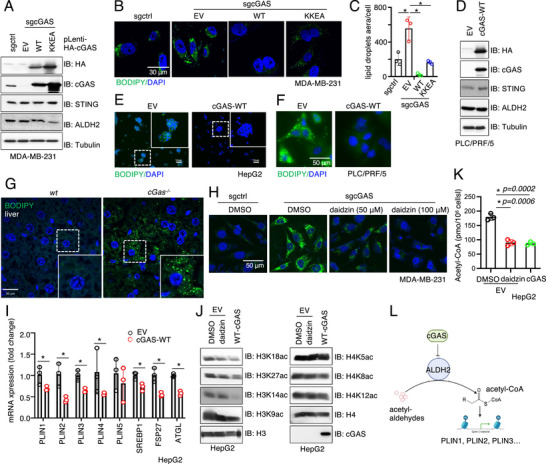
cGAS suppresses ALDH2 activity to restrain lipid droplet formation A) IB analysis of WCL from MDA‐MB‐231 cells depleted of cGAS and re‐expressing either cGAS WT or cGAS KKEA mutant via lentiviral infection. Cells were selected with 2 mg/ml blasticidin for 72 h to eliminate non‐infected cells. B) Representative IF images for lipid droplets staining by BODIPY in the indicated cells. Scale bar = 30µm. C) Quantification of lipid droplets from (B) using ImageJ. Error bars were calculated as mean+/‐SD, n=3 biological replicates. *p < 0.05 (one‐way ANOVA test). D) IB analysis of WCL from control and cGAS‐WT stably expressing PLC/PRF/5 cells. E,F) Lipid droplets were stained with BODIPY in control and cGAS‐WT stably expressing HepG2 and PLC/PRF/5 liver cell lines, respectively. Scale bar = 50µm. G) Livers were harvested from 10‐week‐old WT and cGas ‐/‐ mice, and lipid droplets were stained using BODIPY. Scale bar = 30µm. H) Representative IF images for lipid droplets staining by BODIPY in the indicated cells. Scale bar = 50µm. I) RT‐PCR analysis of indicated mRNA levels in control or cGAS‐WT stably expressing HepG2 cells. Error bars were calculated as mean+/‐SD, n=3 biological replicates. *p < 0.05 (one‐way ANOVA test). J) IB analysis of WCL from indicated HepG2 cells. Where indicated, 10 µM daidzin was used to treat HepG2 cells for 12 h prior to cell collection. K) Acetyl‐CoA ELISA assays to measure cellular acetyl‐CoA levels in indicated HepG2 cells. Where indicated, 10 µM daidzin was used to treat HepG2 cells for 12 h prior to cell collection. Error bars were calculated as mean+/‐SD, n=3 biological replicates. *p < 0.05 (one‐way ANOVA test). L) A cartoon illustration of our proposed model: cGAS suppresses ALDH2 activity to reduce its ability to convert acetyl‐aldehydes into acetate and acetyl‐CoA, which promotes histone acetylation for transcribing enzymes involved in lipid synthesis and lipid droplet formation.

Since depletion of endogenous cGAS increased ALDH2 activity in cells (Figure 2H), we next investigated the cellular effects of this increase. ALDH2 functions as an oxidoreductase that counteracts ROS (oxidative stress)‐induced lipid peroxidation by converting toxic aldehydes into non‐toxic carboxylic acids. Because elevated ROS disrupts intracellular lipid metabolism, we hypothesized that cGAS may regulate lipid metabolism by suppressing ALDH2 activity. Intracellular lipids are largely stored in lipid droplets that are specialized organelles characterized by a core of neutral lipids surrounded by a phospholipid monolayer.^[^ ^60^ ^]^ Lipid droplets are originated from ER and communicate with multiple cellular organelles to control cell physiology including lipid peroxidation, ER stress response, and ROS metabolism.^[^ ^61^, ^62^, ^63^, ^64^ ^]^ Using BODIPY staining to visualize intracellular lipid droplets, we observed that cGAS depletion significantly increased lipid droplet formation in MDA‐MB‐231 cells, whereas re‐expression of either WT‐cGAS or the DNA‐binding‐deficient KKEA‐cGAS effectively reduced lipid droplet levels (**Figure** **4**A–C; Figure S4A,B, Supporting Information). Similarly, introducing cGAS into cGAS‐low liver cancer HepG2 or PLC cells (Figure 4D) significantly reduced lipid droplet levels (Figure 4E,F). Examination of mouse liver tissues from *WT* and *cGas^‐/‐^* C57BL/6 mice revealed comparable liver size and morphology (Figure S4C, Supporting Information), but a significant increase in lipid droplet number in *cGas^‐/‐^* livers (Figure 4G). Importantly, the increase in lipid droplets induced by cGAS depletion could be reversed by either re‐introducing WT‐cGAS (Figure 4B; Figure S4A, Supporting Information) or pharmacologically inhibiting ALDH2 with daidzin (Figure 4H). These data suggest that cGAS regulates lipid droplet content in cells largely through inhibiting ALDH2. Notably, inhibiting cGAS activity by a small molecule inhibitor G140 did not affect lipid droplet levels in cells (Figure S4D, Supporting Information), suggesting the innate immune‐related cGAS activity might not be involved in its regulation of lipid droplets.

To investigate the mechanism(s) underlying cGAS depletion‐induced lipid droplet formation, we examined whether cGAS regulates the expression of genes controlling synthesis of neutral lipids. To this end, we found that re‐expressing cGAS in cGAS‐low HepG2 cells dramatically suppressed transcription of a cohort of key enzymes responsible for neutral lipid synthesis, including PLIN1, PLIN2, PLIN3, PLIN4, SREBP1, FSP27 and ATGL (Figure 4I). Furthermore, cGAS reconstitution, or pharmacological ALDH2 inhibition by daidzin, reduced H3K18ac (enriched in promoters of actively transcribing genes^[^ ^65^ ^]^) and H3K27ac (associated with active enhancers^[^ ^66^ ^]^) markers that earmark active transcription (Figure 4J) in HepG2 cells, suggesting decreased transcriptional activity. This effect may result from ALDH2‐mediated metabolism of acetaldehyde into acetate, which is subsequently converted into acetyl‐CoA by ACSS2 (acetyl‐CoA synthetase 2).^[^ ^67^ ^]^ Indeed, either cGAS expression, or ALDH2 inhibition by daidzin, reduced cellular acetyl‐CoA levels in HepG2 cells (Figure 4K). Therefore, elevated acetyl‐CoA levels resulting from cGAS depletion could enhance histone acetylation, including H3K18ac and H3K27ac. Alternatively, cGAS/ALDH2 governed changes in acetaldehyde may trigger cellular responses such as DNA damage and oxidative stress to influence gene expression. Collectively, our findings suggest that cGAS/ALDH2 signaling may regulate histone acetylation, such as H3ac, to influence neutral lipid synthesis and modulate lipid droplet levels in cells (Figure 4L).

### 2.6 cGAS Depletion‐Induced Lipid Droplet Formation Confers Ferroptosis Resistance

**Figure 5:**
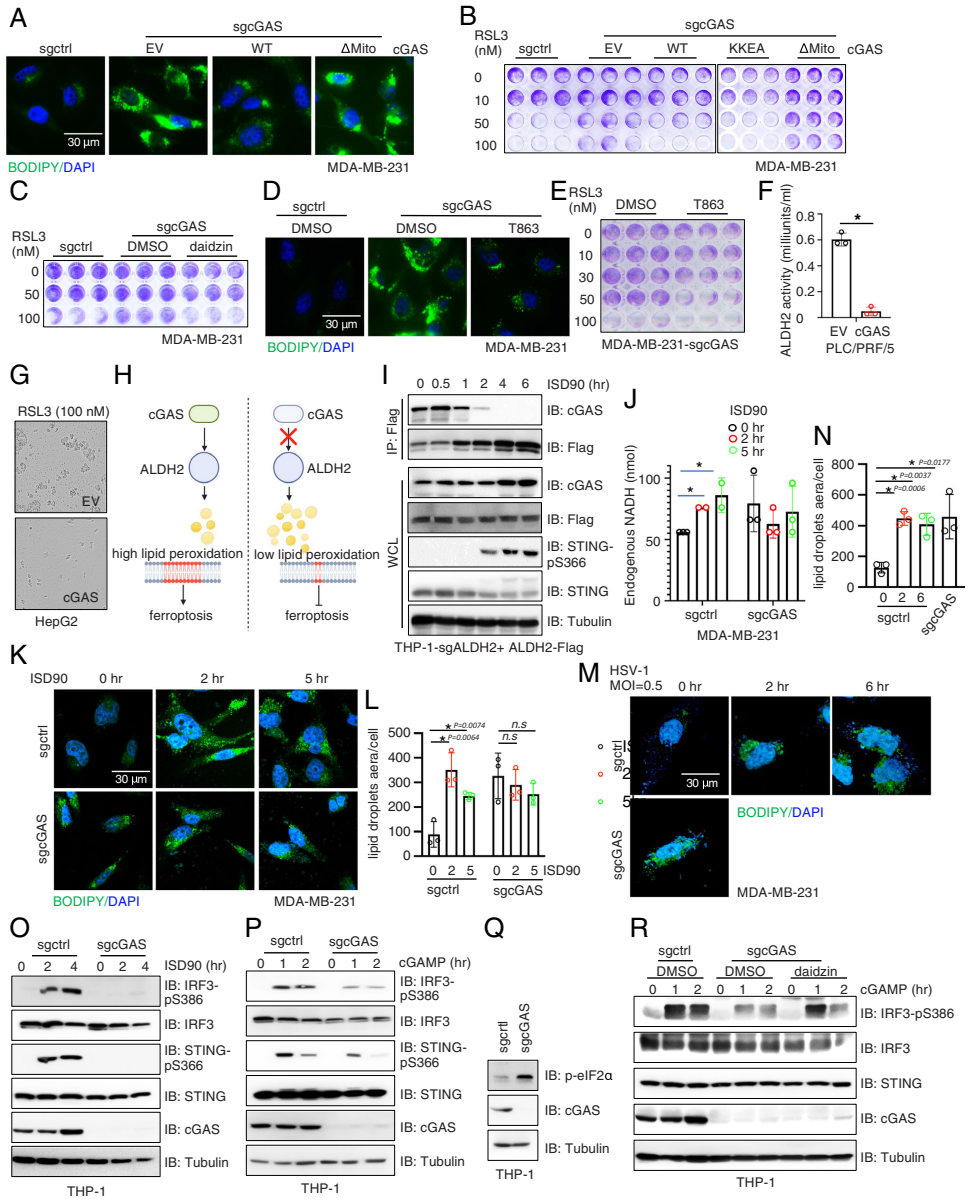
The cGAS/ALDH2 signaling regulates cellular ferroptosis response and STING activation by modulating lipid droplet function A) Representative IF images for lipid droplets staining by BODIPY in indicated MDA‐MB‐231 cells. Scale bar = 30µm. B,C,E) Representative colony formation assays using indicated MDA‐MB‐231 cells treated with indicated doses of RSL3. Where indicated, 50 µM daizin was included or 20 µM T863 was included in the treatments. D) Representative IF images for lipid droplets staining by BODIPY in indicated MDA‐MB‐231 cells. Where indicated, 30 µM T863 was used to treat MDA‐MB‐231‐sgcGAS cells for 24 hrs. Scale bar = 30µm. F) Endogenous NADH levels were measured in control and cGAS‐WT stably expressing HepG2 cells. Error bars were calculated as mean+/‐SD, n=3 biological replicates. *p < 0.05 (one‐way ANOVA test). G) RSL3 at 100 nM concentration was used to induce ferroptosis in control or cGAS‐WT stably expressing HepG2 cells. Images were captured using IncuCyte® Live‐Cell Analysis equipment. H) A cartoon illustration depicting our hypothesis: cGAS suppressing ALDH2 activity, reducing lipid droplet formation, and sensitizing cells to ferroptosis. I) IB analysis of Flag‐IP and WCL from THP‐1 cells depleted of ALDH2 and re‐expressing ALDH2‐WT, treated with ISD90 for the indicated time points. J) Endogenous NADH levels were measured in control or cGAS‐depleted MDA‐MB‐231 cells treated with ISD90 for the indicated time points. Error bars were calculated as mean+/‐SD, n=3 biological replicates. *p < 0.05 (one‐way ANOVA test). K,L) Representative IF images for lipid droplets staining by BODIPY in indicated MDA‐MB‐231 cells transfected with ISD90 (5 µg/ml) for the indicated time points and quantified in (L). Error bars were calculated as mean+/‐SD, n=3 biological replicates. *p < 0.05 (one‐way ANOVA test). Scale bar = 30µm. M) Representative IF images for lipid droplets staining by BODIPY in indicated MDA‐MB‐231 cells infected with HSV‐1 (MOI=0.5) at the indicated time points and quantified in N). Error bars were calculated as mean+/‐SD, n=3 biological replicates. *p < 0.05 (one‐way ANOVA test). Scale bar = 30µm. O,P) IB analysis of WCL from control or cGAS‐depleted THP‐1 cells transfected with 5 µg/ml ISD90 (M) or treated with 5 µg/ml 2′3′‐cGAMP for the indicated time points. Q) IB analysis of WCL from control or cGAS‐depleted THP‐1 cells. R) IB analysis of WCL from control or cGAS‐depleted THP‐1 cells treated with or without Daidzin (50 µM) following cGAMP stimulation at indicated time points.

Ferroptosis is a form of cell death with accumulated iron and lipid peroxidation,^[^ ^68^, ^69^ ^]^ and lipid metabolism has been tightly linked to ferroptosis sensitivity.^[^ ^70^, ^71^ ^]^ Lipid droplets, as major storage sites for PUFA (polyunsaturated fatty acids) and MUFA (monounsaturated fatty acids), have recently been recognized as key modulators of ferroptosis. For example, a recent study demonstrated that cell cycle arrest induces lipid droplet formation to buffer excess extracellular polyunsaturated fatty acids (PUFAs), thereby contributing to ferroptosis resistance.^[^ ^72^ ^]^ We therefore investigated whether cGAS/ALDH2‐mediated changes in lipid droplets influence ferroptosis. Consistent with cGAS suppressing ALDH2 activity, the increase in lipid droplet formation caused by cGAS depletion was reversed by re‐expression of WT‐cGAS or the DNA‐binding‐deficient KKEA‐cGAS (Figure 4B), but not by ΔMito‐cGAS (**Figure** **5**A). As a result, cGAS depletion‐resulted lipid droplet accumulation conferred resistance to the ferroptosis inducer RSL3, which could be reversed by re‐expressing WT‐ or KKEA‐cGAS, but not by ΔMito‐cGAS (Figure 5B; Figure S5A–C, Supporting Information). Furthermore, pharmacologically inhibition of ALDH2 with daidzin also reduced lipid droplet levels in cGAS‐depleted MDA‐MB‐231 cells (Figure 4H), sensitizing these cells to ferroptosis (Figure 5C). These data support the notion that cGAS‐mediated suppression of ALDH2 activity contributes to ferroptosis responses. To further confirm the role of lipid droplets, we treated MDA‐MB‐231 cells depleted of cGAS with a DGAT1 (diacylglycerol acyltransferase) inhibitor T863,^[^ ^73^ ^]^ which blocks triacylglycerol synthesis and lipid droplet formation. This treatment reduced lipid droplets (Figure 5D; Figure S5D, Supporting Information) and sensitized cells to RSL3 (Figure 5E). Similarly, ectopic cGAS expression in cGAS‐low HepG2 and PLC cells inhibited ALDH2 activity (Figures 2J and 5F), reduced lipid droplet levels (Figure 4E,F), and increased sensitivity to RSL3 (Figure 5G). Collectively, these findings suggest that cGAS depletion activates ALDH2, increasing lipid droplet levels and contributing to ferroptosis resistance (Figure 5H).

### 2.7 DNA Challenges Disrupt cGAS‐ALDH2 Interactions, Promoting Lipid Droplet Formation and Impairing STING Signaling

We next explored pathophysiological factors regulating cGAS/ALDH2 signaling and its impact on lipid droplet formation. ISD90 stimulation triggered a dissociation of cGAS from ALDH2 in THP1 (Figure 5I), BPH1 (Figure S5E, Supporting Information) and HepG2 (Figure S5F,G, Supporting Information) cells. Given the potent ability of ISD90 to trigger cGAS activation,^[^ ^23^ ^]^ we speculated that ISD90‐induced relief of ALDH2 suppression by cGAS may contribute to modulation of cGAS/STING activity. Consistent with reduced cGAS binding to ALDH2, ISD90 also significantly induced ALDH2 activity (evidenced by increased endogenous NADH levels, Figure 5J) and subsequently increased lipid droplet levels in MDA‐MB‐231 cells (Figure 5K,L). In contrast, *cGAS*‐depleted cells exhibited elevated basal levels of lipid droplets, and ISD90 did not further promote lipid droplet formation (Figure 5K,L). Notably, infection of MDA‐MB‐231 cells with the DNA virus HSV‐1 similarly induced lipid droplet formation (Figure 5M,N), highlighting the pathological relevance of this process.

Given the critical role of lipid droplets in ER interactions and regulation of ER function,^[^ ^62^, ^74^ ^]^ as well as the requirement of STING trafficking from the ER to ERGIC compartments for its activation^[^ ^75^ ^]^ and termination,^[^ ^76^ ^]^ we next investigated whether DNA challenge‐induced lipid droplet formation plays a role in regulating cGAS/STING signaling. As expected from an essential role of cGAS as a major cytosolic DNA sensor, cGAS depletion nearly abolished the cellular response to ISD90 stimulation (Figure 5O). Surprisingly, treatment with 2′3′‐cGAMP, the major cGAS enzymatic product and a natural STING agonist, which theoretically should activate STING regardless of cGAS, resulted in reduced STING activation in *cGAS*‐depleted THP‐1 cells compared to WT cells, as evidenced by decreased pIRF3 and pSTING signals (Figure 5P), lower transcription of IFN‐β (Figure S5H, Supporting Information), and reduced expression of IFN‐β‐stimulated genes such as CXCL10 (Figure S5I, Supporting Information). cGAS depletion also dampened THP‐1 cell response to the orally available STING agonist MSA2^[^ ^77^ ^]^ (Figure S5J, Supporting Information). These data suggest that DNA insult‐induced lipid droplet formation, likely through the release of cGAS suppression on ALDH2, impairs efficient STING activation.

Given the close association of lipid droplets with ER, we observed that depletion of endogenous cGAS in THP1 cells increased ER stress as evidenced by increased p‐eIF2α signals (Figure 5Q). Ectopic expressing cGAS in cGAS‐low HepG2 cells suppressed p‐eIF2α signals (Figure S5K, Supporting Information). Notably, pharmacologically inhibition of ALDH2 by daidzin (Figure 5R) or blockade of lipid droplet formation by DGAT inhibitor T863 (Figure S5L, Supporting Information), largely rescued cell responses to 2′3′‐cGAMP treatment, highlighting a critical role of cGAS/ALDH2/lipid droplet signaling in regulating STING agonists‐induced STING activation.

### 2.8 cGAS Depletion Facilitates FFA‐Induced Lipid Droplet Accumulation in Liver Cells

**Figure 6:**
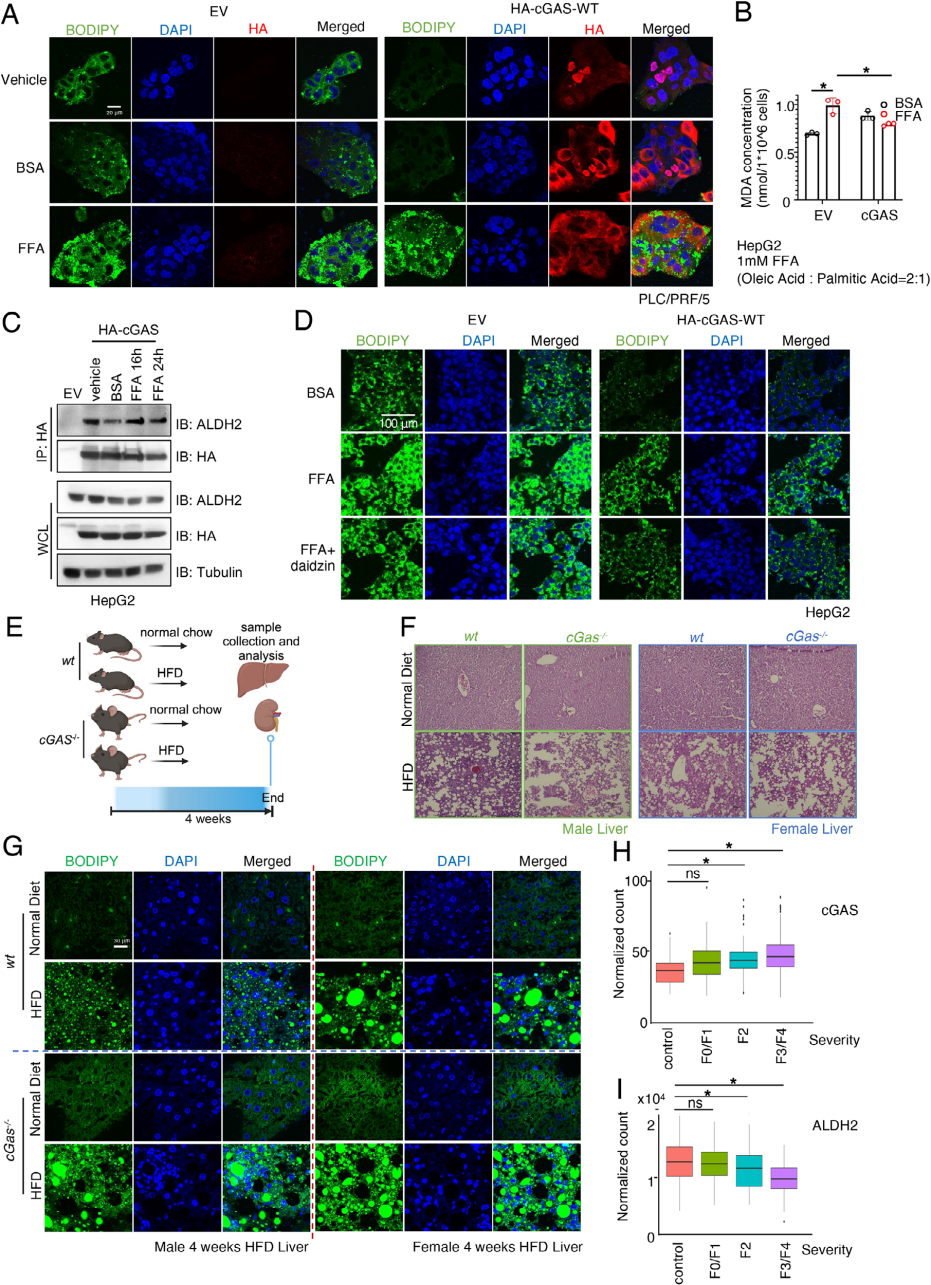
Loss of cGAS promotes MASLD development in liver cells and mouse livers A) Representative IF images for lipid droplets staining by BODIPY in control or cGAS‐WT stably expressing HepG2 cells treated with FFA or BSA control. Scale bar = 20µm. B) Measurements of ALDH2 activity in vehicle control or FFA treated HepG2 cells. Error bars were calculated as mean+/‐SD, n=3 biological replicates. *p < 0.05 (one‐way ANOVA test). C) IB analysis of WCL and HA‐IP from cGAS stably expressing HepG2 cells treated with FFA for the indicated time points. D) Representative IF images for lipid droplets staining by BODIPY in control or cGAS‐WT stably expressing HepG2 cells treated with FFA stimulation with or without daidzin treatment. Scale bar = 100µm. E) A cartoon illustration of the diet‐manipulated animal experiments. Refer to the method section for details.10‐week‐old WT and cGas‐/‐ C57BL/6 mice were treated with normal or HFD, following euthanasia, livers were harvested to perform lipid droplet staining, RT‐PCR, and H&E staining at weeks 4 and 8. F) Representative H&E staining of livers from WT and cGas‐/‐ C57BL/6 mice receiving normal chow or HFD for 4 weeks. G) Representative IF images for lipid droplets staining by BODIPY in livers from WT and cGas‐/‐ C57BL/6 mice receiving normal chow or HFD for 4 weeks. Scale bar = 30µm. H,I) Analyses of MASLD patient cohorts for association of cGAS (H) or ALDH2 (I) mRNA levels with MASLD severity (F0, F1, F2, F3, and F4). *p < 0.05 (one‐way ANOVA test).

Lipid droplets accumulation is associated with a wide range of human disorders,^[^ ^62^, ^74^ ^]^ and its role in the liver is well established. Increased lipid droplets containing neutral lipids (typically TAGs) is a hallmark of hepatic steatosis, commonly referred to as MASLD (metabolic dysfunction‐associated steatotic liver disease, previously known as non‐alcoholic fatty liver disease), which occurs independently of alcohol use or viral infections. More advanced stages of MASLD, characterized by inflammation, hepatocyte injury, or fibrosis, are classified as MASH (metabolic dysfunction‐associated steatohepatitis, previous known as non‐alcoholic steatohepatitis).^[^ ^78^ ^]^ To investigate whether cGAS‐governed lipid droplet formation contributes to MASLD, we treated liver PLC/PRF/5 cells with free fatty acids (FFA; oleic acid:palmitic acid = 2:1), a widely used in vitro MASLD model.^[^ ^79^ ^]^ Ectopic cGAS expression in PLC/PRF/5 cells suppressed FFA‐induced lipid droplet formation (**Figure** **6**A). In contrast, PLC/PRF/5 cells with minimal endogenous cGAS expression exhibited markedly higher basal lipid droplet levels, which were further enhanced by FFA, exceeding levels observed in cGAS‐expressing cells (Figure 6A; Figure S6A, Supporting Information). Similarly, FFA more strongly increased lipid droplet levels in HepG2 cells lacking cGAS expression than in their ectopic cGAS‐expressing counterparts (Figure S6B,C, Supporting Information), supporting a suppressive role of cGAS in FFA‐induced lipid droplet accumulation. Restoration of cGAS expression in PLC cells by inhibiting cGAS promoter hypomethylation with the DNMT inhibitor 5‐azacytidine (Figure S6D, Supporting Information) similarly suppressed FFA‐induced lipid droplet formation (Figure S6D, Supporting Information). Consistent with these observations, FFA increased ALDH2 activity in parental, but not in cGAS‐expressing HepG2 cells (Figure 6B). Notably, unlike ISD90, which disrupts cGAS binding to ALDH2 (Figure 5I), FFA did not affect this interaction (Figure 6C). Therefore, the effect of FFA in increasing lipid droplets is likely independent from cGAS‐governed ALDH2 activity regulation. To further investigate whether ALDH2 activity is critical for FFA‐induced lipid droplet formation, we co‐treated cells with the ALDH2 inhibitor daidzin and FFA. Regardless of cGAS expression, daidzin partially suppressed FFA‐triggered lipid droplet formation in both HepG2 (Figure 6D) and PLC/PRF/5 (Figure S6E, Supporting Information) cells. These data suggest that cGAS/ALDH2 signaling is indispensable for FFA‐induced lipid droplet accumulation in liver cells.

### 2.9 cGas Knockout Facilitates HFD‐Induced MASLD in the Mouse

To investigate the pathophysiological role of cGAS/ALDH2/lipid droplet signaling in the development of MASLD, we used a modified high‐fat diet (HFD) known to induce MASLD in mice^[^ ^80^ ^]^ (Research Diet A06071309: a methionine‐ and choline‐deficient diet with 45 kcal% fat). Age‐matched 8‐12‐week C57BL/6 *WT* and *cGas^‐/‐^* mice were fed either normal chow or the modified HFD for 4 weeks, after which animals were sacrificed (Figure S7A, Supporting Information) and their livers and kidneys (as a negative control) were harvested (Figure S7B, Supporting Information). Notably, livers from male *cGas^‐/‐^* mice receiving the modified HFD were larger than those from their *WT* littermates on the same diet (Figure S7B, Supporting Information). Additionally, both male and female *cGas^‐/‐^* livers displayed visible liver scarring in the HFD groups by H&E staining, which was absent in animals on normal chow (Figure 6F). Lipid droplet accumulation increased in HFD‐treated livers of all mice but was significantly more pronounced in *cGas^‐/‐^* mice compared to *WT* controls (Figure 6G). In contrast, HFD had minimal effects on kidney lipid droplet content in either genotype (Figure S7C,D, Supporting Information). Collectively, these findings indicate that cGAS deficiency may accelerate HFD‐induced MASLD in mice.

### 2.10 Increased cGAS Expression is Observed in Human MASLD Patients

Multiple cohort studies using multiple‐omics have suggested potential biomarkers to identify at‐risk steatohepatitis, such as a combination of four proteins (ADAMTSL2, AKR1B10, CFHR4, and TREM2) from a logistic regression model.^[^ ^81^ ^]^ To explore the clinical relevance of cGAS/ALDH2 signaling in MASLD, we first analyzed a publicly available transcriptome dataset (GSE126848) that included patients with varying degrees of MASLD and healthy controls. Compared with healthy individuals, MASLD patients exhibited significantly lower cGAS mRNA expression (Figure S7E, Supporting Information). We next examined our patient cohort collected at Duke University Medical Center, which included 368 MASLD patients. Given fibrosis severity is used clinically to stage MASLD severity/risk for liver related morbidity and mortality, termed as F0, F1, F2, F3, and F4, our patient data cohorts suggested that no significant difference was observed in F0/F1 patients compared with healthy control individuals, while increased cGAS mRNA levels were observed in F2 and F3/F4 patient cohorts (Figure 6H), associated with decreased ALDH2 mRNA levels (Figure 6I). These findings suggest that as MASLD severity increases, cGAS expression rises while ALDH2 expression declines. This opposing regulation may reflect a compensatory mechanism to counteract disease progression, although further mechanistic studies are warranted.

## 3 Discussion

cGAS is best known as a major cytosolic DNA sensor that activates innate immunity to counter infections.^[^ ^1^, ^2^ ^]^ Innate immunity‐independent function of cGAS is just beginning to be appreciated.^[^ ^82^ ^]^ For example, cGAS has been shown to govern cellular senescence.^[^ ^83^ ^]^ Nuclear cGAS was reported to inhibit the repair of damaged DNA^[^ ^27^, ^84^ ^]^ and regulate DNA replication forks.^[^ ^28^ ^]^ Mitochondrial cGAS has been revealed to suppress ferroptosis through facilitating polymerization of DRP1 (dynamin‐related protein 1).^[^ ^49^ ^]^ In this study, we identify a new mitochondrial role for cGAS, independent of innate immunity: cGAS directly binds to and suppresses ALDH2, thereby restraining lipid droplets formation and function, ultimately protecting livers from HFD‐induced MASLD.

In addition to regulating MASLD, we found that cGAS/ALDH2/lipid droplet signaling also influences cellular response to ferroptosis, presumably by buffering and balancing PUFA/MUFA contents in cells and in lipid droplets, given that ferroptosis is driven by lipid peroxidation and ROS accumulation.^[^ ^85^, ^86^ ^]^ Our finding aligns with a recent report showing cell‐cycle dependent lipid droplet level changes confer to ferroptosis resistance.^[^ ^72^ ^]^ However, because lipid droplets are implicated in various cellular functions, including lipid peroxidation, ER stress response, and ROS metabolism,^[^ ^61^, ^62^, ^63^, ^64^ ^]^ further studies are needed to determine whether cGAS/ALDH2 signaling modulates ferroptosis primarily by detoxifying lipid peroxidation products or by regulating ROS homeostasis.

Lipid droplets are widely recognized as energy storage organelles, but their roles in immune responses are increasingly acknowledged. Recent studies demonstrated that lipid droplets are induced during infections with the Zika virus and HSV‐1 in an EGFR‐dependent manner.^[^ ^87^ ^]^ It has also been reported that the formation of lipid droplets following viral infections enhances IFN expression, thereby promoting antiviral defense and limiting viral replication.^[^ ^87^ ^]^ In our study, we observe that dsDNA disrupts the interaction between cGAS and ALDH2. Additionally, we observed that dsDNA accumulation, whether from HSV‐1 infection or dsDNA transfection, triggered lipid droplet formation, which was absent in cGAS‐deficient cells. Enhanced lipid droplet formation attenuated STING activation, coinciding with elevated ER stress that likely impairs STING trafficking from ER to Golgi, a step critical for its activation. These findings suggest that lipid droplets regulate STING activation indirectly through cGAS/ALDH2 signaling, independent of canonical cGAS–2′3′‐cGAMP–STING signaling.

Notably, a recent preprint study reported that ALDH2 negatively impacts host defense against bacterial infection,^[^ ^88^ ^]^ raising the possibility that cGAS loss‐induced ALDH2 activation may enhance host protection against bacterial infection. This function differs from the canonical role of cGAS in innate immunity, which involves triggering IFN‐β expression and adaptive immune activation. Given that both cGAS and ALDH2 are evolutionarily conserved from bacteria to humans,^[^ ^89^ ^]^ this regulatory mechanism may also be evolutionarily conserved. Notbaly, bacterial cGAS forms ubiquitin‐like conjugates,^[^ ^90^ ^]^ and further investigation is needed to determine whether this unique modification is required for bacterial cGAS recognition and regulation of bacterial ALDH.

No obvious developmental or physiological defects have been reported in whole‐body *cGAS^‐/‐^* mice. Recently, a preprint indicated an increased body size in aged *cGAS^‐/‐^* mice.^[^ ^91^ ^]^ This suggests that cGAS loss alone maybe not strongly drive disease development under baseline conditions. Consistent with this notion, whole‐body *cGas* knockout mice are more susceptible to DNA virus infections,^[^ ^2^ ^]^ underscoring its essential role in antiviral defense. In addition, in tauopathy mouse models, deletion of *cGas* alleviated tauopathy‐induced microglial inflammation, partially restored synapse integrity and plasticity, and improved cognitive deficits.^[^ ^92^ ^]^ These findings highlight a pathological context in which cGAS activation contributes to neuroinflammation and neurodegeneration. Here, we identify a distinct physiological role for cGAS in liver metabolism: HFD induced more severe MASLD phenotypes in *cGAS^‐/‐^* mouse livers compared with *WT* littermates, indicating that cGAS may protect livers from diet‐induced disease progression. Reduced hepatic cGAS expression may therefore predispose individuals to develop MASLD upon western diets. Interestingly, previous studies showed *Sting* deletion ameliorated HFD‐induced MASLD by reforming gut microbiota in mouse models,^[^ ^93^ ^]^ which highlights the biological significance of our findings in suggesting an innate immunity‐independent function of cGAS/ALDH2 signaling in modulating MASLD. Notably, future studies should also assess animal body weight, liver fibrosis scores, and standard blood markers of MASLD in our current mouse models, as well as determine whether ALDH2 inhibition or genetic ablation in hepatocyte‐specific *cGas* knockout mice can reverse HFD‐induced MASLD.

In addition, direct evidence linking ALDH2 to MASLD remains limited. Our cellular results suggest that cGAS depletion elevated ALDH2 activity in liver cells and that treatment with daidzin, an ALDH2 inhibitor, reduces lipid droplet formation and mitigates the effects of cGAS deficiency in liver cell models. These findings provide new insights into the pathogenesis of MASLD and suggest a potential therapeutic target.

There are still some limitations from our study. First, the role of lipid droplets in ferroptosis remains contentious, as they can either promote or inhibit ferroptosis depending on the specific lipid species they harbor.^[^ ^72^, ^85^, ^94^, ^95^, ^96^ ^]^ To further clarify the regulatory mechanisms, lipidomic analyses are essential to characterize the lipid composition within lipid droplets influenced by cGAS/ALDH2 signaling. Additionally, live imaging using fluorescent dyes to track lipid droplet dynamics will help elucidate fatty acid trafficking between the cell membrane and lipid droplets, offering deeper insights into this process. It is important to distinguish between exogenous fatty acid uptake into lipid droplets and endogenous lipid synthesis. Furthermore, although we observed reduced cGAS expression in MASLD patient cohorts compared with healthy controls, the sample size of this cohort was limited. A larger, more diverse patient cohort is necessary to conduct an unbiased analysis and confirm these findings. Furthermore, scRNA‐Seq or spatial‐omics analyses to examine hepatocyte‐specific cGAS/ALDH2 signaling warrant further investigation in both healthy and MASLD patient samples.

## 4 Conclusion 

In summary, our study reveals a novel, innate immunity–independent role of cGAS in binding to and suppressing mitochondrial ALDH2 activity. This suppression inhibits lipid droplet formation and function, with physiological consequences that slow MASLD progression in animal models. These findings suggest that cGAS expression may serve as a prognostic marker in MASLD patients and that ALDH2 inhibition could represent a potential therapeutic strategy for clinical management of MASLD.

## 5 Experimental Section

### Cell Culture

HEK293T, MDA‐MB‐231, BPH1, HepG2, PLC/PRF/5, and EA.hy926 cell lines were cultured in DMEM containing 10% fetal bovine serum (FBS), 100 U mL^−1^ penicillin, and 100 mg mL^−1^ streptomycin (1% P/S) at 37 °C with 5% CO_2_. THP‐1 cells were maintained in RPMI‐1640 medium supplemented with 10% FBS, 100 U/mL penicillin, and 100 mg mL^−1^ streptomycin under similar conditions.

MDA‐MB‐231 cells (RRID: CVCL_0062) were purchased from ATCC (HTB‐26), HEK293T cells (RRID: CVCL_0063) were purchased from ATCC (CRL‐3216), THP1 (RRID: CVCL_0006) and EA.hy926 (RRID number not available) cells were obtained from Dr. Blossom Damania lab (UNC‐Chapel Hill), HepG2 (RRID: CVCL_0027) and PLC/PRF/5 cells (RRID: CVCL_0485) were obtained from Dr. Jesse Raab lab (UNC‐Chapel Hill). Cells were continuously monitored for mycoplasma contamination to ensure they remained free of contamination. These cell lines were chosen for this study because of cGAS and ALDH2 expression in these cell lines, as well as the study focuses on liver cells.

### Plasmids

HA‐cGAS, Flag‐cGAS, ALDH2‐Flag, and ALDH2‐His constructs were generated by inserting cGAS or ALDH2 cDNA into pCDNA3 vectors. The cDNA was amplified via PCR using BglII‐F and XhoI‐R primers or BamHI‐F and SalI‐R primers, respectively. To create pLenti‐blast‐cGAS and pLenti‐blast‐Sa. ALDH constructs, cGAS or Sa. ALDH cDNA was cloned into the pLenti‐CMV‐HA‐blast vector (Addgene #125133) using BamHI and SalI restriction sites. pLenti‐puro‐cGAS‐sgRNA and pLenti‐puro‐ALDH2‐sgRNA constructs were prepared by inserting annealed sgRNA oligos into the pLenti‐CRISPR‐V2 vector (Addgene #78852), following Feng Zhang lab's BsmBI‐based cloning protocol: (https://media.addgene.org/cms/files/Zhang_lab_LentiCRISPR_library_protocol.pdf).

### Transfection and Infection

The cells were seeded in a 6 cm dish one day before plasmid transfection. Cell transfection was performed using Lipofectamine 3000 or polyethylenimine (PEI), as described previously.^[^ ^97^, ^98^ ^]^

### Lentiviral Transduction

Packaging of lentiviral shRNA or cDNA expressing lenti‐viruses, as well as subsequent infection of various cell lines were performed according to the protocols described previously.^[^ ^97^, ^98^ ^]^ Briefly, lentiviral packaging was performed using 293T cells, which were transfected with the required expression plasmid, pMD2.G, and psPAX2 at a 2:1:1 ratio using PEI. The transfection mixture was prepared in Opti‐MEM. After 24 h, the medium was replaced with fresh DMEM supplemented with 10% FBS and 1% P/S, and the cells were incubated at 37 °C with 5% CO_2_ to facilitate lentivirus production.

Lentiviral supernatants were collected at 48‐ and 72‐h post‐transfection, filtered through a 0.45 µm filter, and supplemented with 10 µM polybrene. Target cells were then cultured in the virus‐containing medium for 8 h, after which the medium was replaced with fresh viral supernatant. Following an additional 24‐h incubation, the medium was exchanged for DMEM or RPMI‐1640 containing 10% FBS and 1% P/S.

Infected cells were allowed to recover in fresh medium for at least 24 h before selection with either 1 µg mL^−1^ puromycin or 5 µg mL^−1^ blasticidin, depending on the viral vector used. After 72 h of selection, Western blot analysis was performed to assess the efficiency of lentiviral transduction.

### Protein Expression and Purification

The codon‐optimized (Escherichia coli expression) full‐length human ALDH2 gene was synthesized from GENEWIZ and subcloned into a pET28a vector containing a His6 tag at the N‐terminus. hALDH2 was expressed in E. coli Rosetta (DE3) cells (Sigma) that were induced with 0.3 mM isopropyl‐1‐thio‐β‐d‐galactopyranoside (IPTG) at optical density at 600 nm (OD600) = 0.6 at 21  °C overnight. Cells were harvested and sonicated in buffers containing 50 mM Tris‐HCl, pH 8.0, 500 mM NaCl, 5% glycerol, 5 mM β‐mercaptoethanol, 1 mM PMSF and 10ug of Benzonase. The lysate was cleared by centrifugation and incubated with Ni Sepharose (Qiagen). The bound protein was eluted with buffer containing 200 mM imidazole and was further purified Q column (GE healthcare) and polished by size‐exclusion chromatography (Superdex 200 Increase 10/300, GE Healthcare) in a buffer containing 1X PBS and 1 mM β‐mercaptoethanol. This was done to isolate what was likely apo hALDH2. Separately another polishing run was performed on the same column with hALDH2 after incubating 2 h in 4 °C with 1 mM NAD+, it was then run in a 1X PBS, 1 mM β‐mercaptoethanol, and 10 µM NAD+ buffer over the same SEC column. This was done to isolate only tetrameric hALDH2.

The codon‐optimized (Escherichia coli expression) full‐length mouse cGAS genes were synthesized from GENEWIZ and sub cloned into a pETSUMO vector containing a His6 tag at the N‐terminal of the SUMO tag. The mcGAS protein was expressed in BL21 (DE3) cells (Sigma) that were induced with 0.6 mM isopropyl‐1‐thio‐β‐d‐galactopyranoside (IPTG) at optical density at 600 nm (OD600) = 0.7 at 19 °C overnight. Cells were harvested and sonicated in buffers containing 50 mM Tris‐HCl, pH 7.4, 600 mM NaCl, 10% glycerol, 5 mM β‐mercaptoethanol,1 mM PMSF, and 10ug of Benzonase. The lysate was cleared by centrifugation and incubated with Ni Sepharose (Qiagen). The bound protein was eluted with buffer containing 400 mM imidazole. Protein was then cleaved using ULP1 sumolase overnight at 4 °C, before being reloaded onto beads to remove the His‐tagged N‐terminal SUMO region. The cleaved mcGAS was further purified by combined Q column and heparin column (GE healthcare) and polished by size‐exclusion chromatography (Superdex 200 Increase 10/300, GE Healthcare) in a buffer containing 25 mM Tris‐HCl, pH 7.4, 100 mM NaCl, and 1 mM Dithiothreitol (DTT). This would ensure that the FL mcGAS was free of any nucleic acids or other contaminants.

### Size‐Exclusion co‐Migration Assay

The hALDH2‐mcGAS complexes were first formed by incubated at a 1:4 molar ration in a buffer containing 1X PBS, 1 mM β‐mercaptoethanol, 1 mM NAD+ at room temperature for 1 h. Samples were then spun down at 12,000 rpm before being applied to a Superdex 200 Increase 10/300 (GE Healthcare), and ran using a buffer containing 1X PBS, 1 mM β‐mercaptoethanol, 10 µM NAD+. Peak fractions were analyzed by denaturing PAGE to visualize monomeric components of each fraction, and approximate molecular weights were determined using a protein standard (BioRad).

### Mass Photometry

All proteins were diluted with fresh and degassed buffer of 1X PBS with 1 mM β‐mercaptoethanol. A standard protein solution in 1X PBS was used daily for calibrating the contrast intensity to mass values. MP measurements were performed using a TWOMP (Refeyn Ltd, Oxford, UK) at room temperature. Microscope slides (24 × 50 mm, 170 ± 5 µm, No. 1.5H, Paul Marienfeld GmbH & Co. KG, Germany) were cleaned by light sonication in a water bath while submerged in milli‐Q water, then isopropanol, and again in milli‐Q water; they were then dried using a pressurized air stream. Six‐well reusable silicone gaskets (CultureWellTM, 50–3 mm DIA x 1 mm Depth, 3–10 µL, Grace Bio‐Labs, Inc., Oregon, USA) were carefully cut and assembled on the cover slide center. After being placed in the mass photometer, and before each acquisition, an 18 µL droplet of PBS was put in a well to enable focusing on the glass surface. Samples were prepped the day before experiments were run, and kept at 10uM concentrations separately at 4 °C. Samples were then diluted the day of to the desired concentrations of between 10 nM–400 nM using aforementioned degassed buffer before combining as necessary for each experimental run. MP data was acquired with a 5‐fold frame averaging below 1 µM protein concentration (Refeyn Ltd). The resulting video data was analyzed using DiscoverMP software (Refeyn, Oxford, UK). Raw contrast values were converted to molecular mass using a standard mass calibration, and binding events combined in 5 kDa bin width. Binding events below 25 kDa were indistinguishable from background. Detection settings were adjusted according to the specific visualization requirements and with a background reading of buffer alone.

### Immunoblot and Immunoprecipitation Analyses

Cells were washed with ice‐cold PBS and lysed in Triton X‐100 buffer (50 mM Tris, pH 7.5; 120 mM NaCl; 1% Triton X‐100) supplemented with protease inhibitors (APExBIO, Cat. No. K1008) and phosphatase inhibitors (APExBIO, Cat. Nos. K1015‐A and K1015‐B) at 4 °C for 10 min. The lysates were then centrifuged at 15,000 rpm for 10 min at 4 °C to remove insoluble debris. Protein concentrations were determined using a NanoDrop OneC spectrophotometer (Thermo Scientific) in combination with the Bio‐Rad protein assay reagent (Cat. No. 5000006).

Equal amounts of protein lysates were separated on 10% SDS‐PAGE gels using SDS‐PAGE running buffer and electrophoresed at a constant voltage of 128 V for 65 min. Proteins were subsequently transferred onto a PVDF membrane using the Bio‐Rad transfer system at 300 mA for 2 h at 4 °C. The PVDF membrane was blocked in 5% non‐fat milk in TBST buffer for 30 min at room temperature and incubated with primary antibodies overnight at 4 °C with gentle shaking. The next day, the membrane was washed three times with TBST (10 min per wash) and incubated with HRP‐conjugated secondary antibodies diluted in 5% non‐fat milk in TBST for 1 h at room temperature with shaking. After three additional 10‐min washes in TBST, the membrane was incubated with ECL reagents for 5 min at room temperature before imaging using a QUANT imager.

For immunoprecipitation of tagged proteins, at least 1 mg of lysate was incubated with agarose beads conjugated to tag‐specific antibodies for 3 h at 4 °C. Beads were washed three times with NETN buffer (20 mM Tris, pH 8.0; 100 mM NaCl; 1 mM EDTA; 0.5% NP‐40), and bound proteins were resolved by SDS‐PAGE, followed by immunoblotting with the indicated antibodies.

For endogenous ALDH2 immunoprecipitation, THP‐1 whole‐cell lysates were incubated with an ALDH2‐specific antibody overnight at 4 °C with gentle shaking. The following day, protein A/G beads were added to the antibody‐lysate mixture and incubated for 1 h at 4 °C with rotation. The beads were then washed three times with NETN buffer, and immunoprecipitated proteins were analyzed by immunoblotting.

### Immunofluorescence and Confocal Analyses

Cells were seeded onto glass coverslips and incubated for 24 h. Afterward, the media was removed, and the cells were washed three times with PHEM buffer (stock solution for 500 ml 2x PHEM: 18.14 g PIPES, 6.5 g HEPES, 3.8 g EGTA, 0.99 g MgSO_4_; pH adjusted to 7.0 with KOH). Fixation was performed using 4% paraformaldehyde (PFA) in PHEM buffer for 10 min at room temperature. Following fixation, cells were washed three times with PHEM buffer. To block and permeabilize the cells, they were incubated with a blocking/permeabilization solution (0.01% saponin, 3% bovine serum albumin (BSA), 10% goat serum, and 300 mM glycine in PHEM buffer) for 40 min at room temperature with rotation. After three additional washes with PHEM buffer, primary antibodies were applied (anti‐cGAS: 1:500, anti‐ALDH2: 1:500, anti‐HA: 1:1000) and incubated overnight at 4 °C with gentle rotation. The following day, cells were washed three times for 10 min each with PHEM buffer, and secondary antibodies were added. For lipid droplet staining, BODIPY 493/507 was included, and the samples were incubated for 1 h at room temperature, protected from light. Cells were then washed three times with PHEM buffer and mounted using ProLong Gold Antifade Reagent with DAPI for nuclear staining. Images were captured using an Olympus VP‐1000 confocal microscope or a KEYENCE BZ‐X710 fluorescence microscope.

### RT‐PCR

Total RNA was extracted using the RNeasy Mini Kit (QIAGEN) and quantified with a NanoDrop OneC spectrophotometer (Thermo Scientific). Complementary DNA (cDNA) synthesis was performed using the iScript™ Reverse Transcription Supermix (Bio‐Rad, Cat. #1708841) according to the manufacturer's instructions. Quantitative reverse transcription PCR (RT‐qPCR) was performed using the ViiA™ 6 Real‐Time PCR System. cDNA templates were mixed with iTaq™ Universal SYBR Green Supermix (Bio‐Rad, Cat. #1725122), and amplification was carried out under the following cycling conditions: (step 1: 95 °C, 10 min; step 2: 40 cycles of 95 °C, 15 s and 60 °C, 1 min; step 3: 95 °C, 15 s; step 4: 60 °C, 1 min). The mRNA expression levels of IFN‐β, CXCL10, PLIN1, PLIN2, PLIN3, PLIN4, PLIN5, SERBP1, FSP27, and ATGL were normalized to U6 expression and analyzed using the comparative Ct (Livak 2^‐ΔΔCt^) method.

### IFN‐β ELISA

Mouse IFN‐β levels in tissue supernatants were measured using an enzyme‐linked immunosorbent assay (ELISA). A high‐binding 96‐well plate was coated with 100 µL of reconstituted capture antibody (4 µg/mL) and incubated overnight at room temperature.

The following day, the plate was washed three times with wash buffer (0.05% Tween‐20 in PBS, pH 7.2–7.4). Residual wash buffer was removed by inverting the plate and tapping it on clean paper towels. The wells were then blocked with blocking buffer (1% BSA in PBS, pH 7.2–7.4) for 1 h at room temperature. The IFN‐β standard was reconstituted in blocking buffer, and 2‐fold serial dilutions were prepared starting from 500 pg mL^−1^. Following the blocking step, standards and tissue supernatant samples were added to designated wells and incubated at room temperature for 2 h. After incubation, the plate was washed three times, and 250 ng mL^−1^ of reconstituted detection antibody was added to each well, followed by a 2‐h incubation at room temperature. The plate was then washed three times, and diluted streptavidin‐HRP was added and incubated in the dark for 20 min. Following another three washes, substrate solution was added to each well. The color change was closely monitored, and the stop solution was added once the desired color intensity was achieved. Absorbance was measured at 450 nm using a microplate reader. For sample preparation, tissue samples were homogenized before supernatant collection. IFN‐β concentrations were expressed as pg/mL ± SD and calculated using a standard curve generated with recombinant mouse IFN‐β protein standard included in the ELISA kit (R&D Systems, Cat. #DY8234‐05).

### Acetyl‐Coenzyme A Assay

The Acetyl‐CoA assays were performed using the Acetyl‐Coenzyme A Assay kit (MAK566, Sigma‐Aldrich) and followed its instructions. Briefly, indicated cells were washed with PBS, then harvested and homogenized by using a glass Dounce homogenizer on ice in PBS, then the protein samples were deproteinized by PCA (Perchloric Acid) precipitation, and the PH was adjusted equals 7.0. 10 µL of each sample were added into triplicate wells of a 96 well plate and brought to a final volume of 50 µL with assay buffer. Reaction Mixture contains assay buffer, substrate mix, enzyme mix was added to each of the well, and mixed by horizontal shaker followed by 30 °C incubation for 5 min. Then 50 µL reaction initiator was added to each well. The fluorescence intensity was measured by BioTek Cytation 5 Cell Imaging reader (λex /λem = 340/460 nm).

### Bacteria Strains

S. aureus (MRSA) USA300 strain was kind gift from Dr. Brian Conlon (UNC‐Chapel Hill). Cells were grown on Tryptic Soy Broth Agar plates (TSA) overnight at 37 °C, then the fresh S. aureus colonies were inoculated into Tryptic soy Broth (TSB) overnight in 37 °C shaker for further assays.

### Protein Interactome Analysis‐Sample Preparation

A 1 L of USA300 S. aureus culture (OD600 ≈1.0) was prepared, and the bacterial pellet was collected by centrifugation. The bacterial pellet was resuspended in 10 mL binding buffer (50 mM Tris‐HCl, pH 8.0, 5 mM imidazole, 100 mM NaCl, 0,1 mM EDTA, with protease inhibitors/phosphatase inhibitors) and transfer to a 50 mL conical tube. After sonication the milky bacteria solution becomes opaquer, and the lysate was transferred to a high‐speed 30 mL centrifuge tube. The lysate was centrifuged at 15,000 xg for 30 min, and the supernatant was transferred into another 30 mL tube. Prewash Ni‐NTA beads were pre‐washed with 1x PBS 3 times at 200 x g. 25 uL Ni‐NTA beads (50% slurry) were incubated with 25 ug His‐cGAS recombinant proteins for 2 hr at 4 °C. Cleared *S. aureus* bacterial lysates were mixed with Ni‐NTA beads pre‐bound with his‐cGAS proteins in a 15 mL tube and rotated at 4 °C for 4 hrs. Beads were washed 3 times using wash buffer (50 mM Tris‐HCl, pH8.0, 20 mM Imidazole (pH8.0), 100 mM NaCl, and o.1 mM EDTA). Bound proteins were eluted by elution buffer (50 mM Tris‐HCl, pH8.0, 300 mM Imidazole (pH8.0), 50 mM NaCl and 0.1 mM EDTA), were subjected to SDS‐PAGE and stained with coomassie. Lanes (1 cm) for each sample (n = 2) were excised, and the proteins were reduced with 5 mM DTT for 30 min at 55C, alkylated with 15 mM IAA for 45 min in the dark at room temperature, and in‐gel digested with trypsin overnight at 37 °C. Peptides were extracted, desalted with C18 spin columns (Pierce), and dried via vacuum centrifugation. Peptide samples were stored at −80 °C until further analysis.

### LC‐MS/MS Analysis

Each sample was analyzed in duplicate by LC‐MS/MS using an Easy nLC 1200 coupled to a QExactive HF (Thermo Scientific). Samples were injected onto an Easy Spray PepMap C18 column (75 µm id × 25 cm, 2 µm particle size) (Thermo Scientific) and separated over a 90 min method. The gradient for separation consisted of 5%–45% mobile phase B at a 250 nl/min flow rate, where mobile phase A was 0.1% formic acid in water and mobile phase B consisted of 0.1% formic acid in 80% ACN. The QExactive HF was operated in data‐dependent mode where the 15 most intense precursors were selected for subsequent HCD fragmentation. Resolution for the precursor scan (m/z 350–1700) was set to 60,000, while MS/MS scans resolution was set to 15,000. The normalized collision energy was set to 27% for HCD. Peptide match was set to preferred, and precursors with unknown charge or a charge state of 1 and ≥ 7 were excluded.

### Data Analysis

Raw data files were processed using Proteome Discoverer version 2.1 (Thermo Scientific). Peak lists were searched against a Uniprot Staphylococcus aureus database (ID: 46170, downloaded March 2019, containing 42779 sequences) appended with a common contaminants database. The following parameters were used to identify tryptic peptides for protein identification: 10 ppm precursor ion mass tolerance; 0.02 Da product ion mass tolerance; up to two missed trypsin cleavage sites; carbamidomethylation of Cys was set as a fixed modification; oxidation of was set as a variable modification.

Scaffold (version 4.7.3, Proteome Software) was used for further analysis. Peptide identifications were accepted if they could be established at greater than 95% probability to achieve an FDR less than 0.1% by the Scaffold Local FDR algorithm. Protein identifications were accepted if they could be established at greater than 99.0% probability and contained at least 2 identified peptides. Relative quantitation was performed using the calculated quantitative values (Spectral Counts) within Scaffold.

### ALDH2 Enzymatic Activity Measurement

ALDH2 activity was measured using the Aldehyde Dehydrogenase Activity Colorimetric Assay Kit (Sigma‐Aldrich, Cat. #MAK082) according to the manufacturer's instructions. Briefly, cells were seeded one day prior to the ALDH2 activity measurement. Subsequently, 1 × 10^6 cells were homogenized in 200 µL of the ALDH assay buffer provided in the kit. The homogenates were then centrifuged at 13,000 g for 10 min to remove insoluble material. To generate a standard curve, NADH standards were prepared by diluting them in the ALDH assay buffer to obtain concentrations of 0, 2, 4, 6, 8, and 10 nmol per well. A sample blank was included for each sample to account for NADH background interference. The reaction mixtures were prepared using the ALDH assay buffer, ALDH substrate mix, and acetaldehyde in the specified ratios recommended in the kit. Each well of a clear 96‐well plate was loaded with 50 µL of the sample, sample blank, or standard. An equal volume (50 µL) of the appropriate reaction mix was then added to each well. The plate was covered, protected from light, and incubated at room temperature with gentle horizontal shaking for 5 min. Absorbance at 450 nm was recorded every 2 min for 2 h or until the most active sample exceeded the highest standard value. ALDH2 activity was expressed in milliunits per milliliter (mU/mL) and calculated based on the NADH standard curve.

### Lipid Peroxidation Assay

The lipid peroxidation assay was performed using the Lipid Peroxidation (MDA) Assay Kit (Colorimetric/Fluorometric) (Abcam, Cat. #ab118970) following the manufacturer's protocol. Briefly, 2 × 10^6 cells were homogenized in 303 µL of lysis buffer (300 µL MDA lysis buffer with 3 µL BHT stock) using a Dounce homogenizer on ice. The samples were then sonicated to shear DNA and reduce solution viscosity, followed by centrifugation at 13,000 g for 10 min to remove insoluble material. For the reaction, 600 µL of Developer VII/TBA reagent was added to Eppendorf tubes containing 200 µL of either the sample or MDA standards. The tubes were incubated at 95 °C for 1 h and then cooled in an ice bath for 10 min. After incubation, 200 µL of the reaction mix was transferred to a clear 96‐well plate, and absorbance was measured at 532 nm using a microplate reader. MDA concentration was expressed in nmol/mL and calculated using the MDA standard curve.

### Aldehyde Measurement

Aldehyde levels were measured using the Fluorometric Aldehyde Assay Kit (Sigma‐Aldrich, Cat. #MAK141) following the manufacturer's protocol. For sample preparation, mouse blood was collected after oral gavage with 5 mg/kg alcohol and allowed to clot at room temperature for 30 min. The samples were then centrifuged at 1,500 g for 10 min, and the serum was carefully transferred to a clean tube. Aldehyde standards were prepared by performing a three‐fold serial dilution, starting from 1,000 µM, using the assay buffer provided in the kit. The master reaction mix was prepared by diluting it in assay buffer, and 50 µL of the master reaction mix was added to the standard, blank control, and sample wells in a black 96‐well plate. The plate was then covered, protected from light, and incubated at room temperature on a horizontal shaker for 15–30 min. After incubation, 25 µL of reaction buffer was added to each well, and fluorescence intensity was measured (λ_ex_ = 365 nm / λ_em_ = 435 nm). Aldehyde concentrations were calculated using the aldehyde standard curve.

### NADH Measurement

NADH levels were measured using the NAD/NADH Cell‐Based Assay Kit (Cayman, Cat. #600480) according to the manufacturer's protocol. Briefly, 10^4 cells were seeded in a 96‐well plate one day before the assay. On the day of the experiment, the cell culture medium was removed. For THP‐1 cells, the plate was centrifuged at 500 g for 5 min before medium removal. The cells were then washed with 120 µL of assay buffer, followed by centrifugation at 500 g for 5 min. After carefully aspirating the assay buffer, 110 µL of permeabilization buffer was added to each well, and the plate was incubated at room temperature for 30 min with gentle shaking. Following incubation, the plate was centrifuged at 1,000 g for 10 min at room temperature. NADH standard was 2‐fold serial diluted using permeabilization buffer. The supernatants from the samples, along with the prepared standards, were transferred to a new clear 96‐well plate. Then, 100 µL of reaction solution was added to each well using a multichannel pipette, and the plate was incubated on an orbital shaker for 90 min at room temperature. Absorbance was measured at 450 nm, and NADH levels were calculated based on the NADH standard curve.

### Frozen section Hematoxylin and Eosin (H&E) Staining

The slides were washed three times with PBS at room temperature, followed by rinsing with a gentle stream of tap water to remove OCT. The slides were then stained with Hematoxylin for 30 s and rinsed with tap water for 1 min. Next, the slides were sequentially dipped in 70% and 95% ethanol for 30 s each, and counterstained with Alcoholic‐Eosin for 1 min. To dehydrate the slides, they were treated with two changes of 95% ethanol and three changes of 100% ethanol, each for 15 s. Finally, the slides were cleared using two changes of Xylene, each for 1 min, and mounted with a coverslip using mounting solution. Images were captured using the KEYENCE BZ‐X710 microscope.

### Gel Filtration Chromatography

The Superdex‐200 size exclusion column is initially cleaned by flushing with 1 column volume (CV) of 0.5 m KOH, followed by rinsing with 2 CVs of deionized (DI) water. The column is then stored in 1X PBS solution without a reducing agent. To verify the column's separation performance, it is calibrated using Cytiva standard low molecular weight (LMW) or high molecular weight (HMW) calibration kits and blue dextran. Calibration begins by equilibrating the column with two CVs of calibration buffer. After equilibration, the calibration standard is injected, and two CVs of calibration buffer are eluted. The elution volume for each peak is recorded. A standard curve is generated by plotting the logarithm (log10) of the molecular weight of each protein standard against the ratio of its elution volume (Ve) to the blue dextran void volume (V0), adjusted by the total column volume (Vc), using the equation:

(1) $$K=\frac{V_{e}-V_{0}}{V_{c}-V_{0}}$$

Following calibration, the column is washed with 2 CVs of DI water and equilibrated with 2 CVs of 1X PBS, making it ready for use. Prior to any experiment, the column is equilibrated with 2 CVs of elution buffer. All buffers and samples are filtered through a 0.22 µm filter, and any samples are centrifuged to remove debris or aggregates. Elution is performed using 1.5 CVs of elution buffer. The ratio of the elution volume to the void volume is calculated, and the standard curve is used to estimate the molecular size of the eluted species.

### FFA Treatment

For 150 mM Palmitic acid (PA) stock solution, 41.8 mg of PA was weighed and dissolved by adding 1 mL of 50% (v/v) ethanol, followed by heating at 65 °C, with periodic vortexing. Similarly, for 150 mM Oleic acid (OA) stock solution, 45.7 mg of OA was weighed, and 500 µL of 100% ethanol was added. The solution was heated at 65 °C for 15 min. Afterward, 500 µL of MilliQ water was added, and the solution was vortexed, then returned to 65 °C until further use. To prepare a 10% fatty acid‐free BSA solution, 5 g of BSA was dissolved in 50 mL of MilliQ water, filtered through a 0.22 µm filter, and stored at 4 °C. For 1 mM fatty acid working solution (PA: OA = 2:1), 134 µL of the 10% BSA solution was added to a 1.5 mL tube and heated in a 37 °C water bath for 5 min. Then, 4.4 µL of OA and 2.2 µL of PA were added, and the mixture was returned to the water bath at 37 °C for 1 h. Subsequently, 866 µL of culture medium (DMEM) was added and heated to 37 °C. For the control group, 6.6 µL of 50% ethanol was added to 134 µL of 10% BSA and processed as described above. Finally, The FFA‐supplemented culture medium was added to cells 24 h after seeding and incubated at 37 °C with 5% CO2 for overnight, while an equal volume of control solution was added to the vehicle‐treated cell group.

### Mouse Experiments

Mice were maintained in a specific‐pathogen‐free facility at the University of North Carolina (UNC) at Chapel Hill. This study utilized wild‐type C57BL/6J mice (Jackson strain #000664) and *cGas^‐/‐^* C57BL/6J mice (Jackson strain #026554). All mouse experiments were reviewed and approved by the UNC Institutional Animal Care and Use Committee (IACUC) under protocol #25‐017.0.

### S. Aureus Infection Model

A 3 × 4 cm area of fur on the back of 8–12‐week‐old WT and *cGas^‐/‐^* C57BL/6J mice was shaved. Subsequently, 2 x 10^7^ CFU USA300 MRSA was subcutaneously injected into the shaved area. The mice were observed for 2–3 h post‐injection to ensure survival. Skin lesion size and body weight were monitored and recorded daily. Mice were euthanized between day 1 and day 3 post‐infection. After euthanasia, the skin was carefully nicked with sterile scissors and excised. The excised skin was cut into ≈2 mm sections and placed in PBS. Additionally, organs including the liver, kidneys, and spleen were harvested for CFU determination. This animal experiment is approved under IACUC # 24‐029.

### CFU Determination

Following euthanasia, the livers, skin, kidneys, and spleens were immediately harvested from the mice and placed in 1 mL of PBS. The organs were then weighed, homogenized, and diluted at a 1:10 ratio in PBS. The resulting solutions were prepared in 96‐well plates, and 40 µL from each dilution (distributed as 4 × 10 µL aliquots) was plated onto Tryptic Soy Agar plates. The plates were incubated overnight at 37 °C for subsequent analysis.

### Mouse Ethanol Oral Gavage Assays

Ethanol or water (control) was administered via oral gavage at a concentration of 5 g kg^−1^ to 8–12‐week‐old wt and *cGas^‐/‐^* C57BL/6J mice. 6 h after ethanol administration, the mice were euthanized, and blood was collected following rapid eyeball removal. Livers were harvested, rinsed in PBS, and fixed in paraformaldehyde for histological analysis.

### Mouse HFD Model

Eight‐week‐old mice were fed either a high‐fat diet (HFD; Research Diet A06071309, a methionine‐ and choline‐deficient diet with 45 kcal% fat) or a normal diet for 4 or 8 weeks. The mice were housed under a 12‐h light/dark cycle, with food consumption and body weight monitored and recorded every two days. At the end of the feeding period, the mice were euthanized, and the livers and kidneys were immediately harvested and rinsed in PBS. The shape and size of the organs were documented before fixation in paraformaldehyde for lipid droplet staining and histological analysis.

### RNA‐Seq Analysis of MASLD Patient Cohort

The Duke MASLD cohort (GSE213623) data consists of 368 samples, including 69 patients with obesity but no MASLD (n = 69) and those with obesity and biopsy‐proven MASLD (n = 299). Raw fastq files were aligned to the hg38 genome using STAR v2.7.4, and a raw count matrix was generated using Subread. DESeq2 (v 1.38.3) was used to filter low count genes and construct a normalized count matrix which was used as input for the boxplots using ggplot2 in R (v 4.4).

### Statistical Analysis

No data exclusion was performed for statistical analysis. Error bars were calculated as mean+/‐SD, and n = biological replicates are indicated in each figure legends. Statistical comparisons between the control and experimental groups were performed using mostly one‐way ANOVA as indicated in figure legends. All analyses were conducted using GraphPad Prism version 8 (San Diego, CA, USA), and a p‐value of ≤ 0.05 was considered statistically significant.

## Conflict of Interest

The authors declare no conflict of interest.

## Author Contributions

Y.W., and Y.D. contributed equally to this work. Y. W., Y. D., and P.L. conceived, supervised, and acquired fundings for this project. Y.W., Y.D., J.C., Q.H., Z.Z, Y.Z. S.E.R. and L.L. performed the experiments. Y.W., Y.D., J.C., Q.H., D.U., Z.Z, Y.Z., and S.E.R. were engaged in final data analysis and visualization. Y.W. guided data analysis. A.S.B., B.C., E.A.M., B.D., A.M.D., and P.L. supervised the study. Y.W. and P.L. wrote the manuscript with input from all authors. All authors were involved in manuscript revising and gave the final approval of the manuscript.

## Materials Availability

Materials generated from this study can be requested to the lead contact, Pengda Liu (pengda_liu@med.unc.edu).

## Supporting information



Supporting Information

Supporting Information

## Data Availability

All data needed to evaluate the conclusions in the paper is present in the paper and/or the Supplementary Materials. The data that support the findings of this study are available from the corresponding author upon reasonable request.
