# Supplementary material for: cGAS Inhibits ALDH2 to Suppress Lipid Droplet Function and Regulate MASLD Progression
Source: Adv Sci (Weinh). 2025 Oct 3;12(46):e08576. doi: 10.1002/advs.202508576 (PMC12697864; doi:10.1002/advs.202508576)
Supplement: Supplementary file 1 — Supporting Information [file ADVS-12-e08576-s001.pdf]

Supplemental information

cGAS Inhibits ALDH2 to Suppresses Lipid Droplet Function and Regulate MASLD Progression

Ying Wang, Yu Deng, Jianfeng Chen, Quentin Hahn, David S. Umbaugh, Zhigang Zhang, Yanqiong Zhang, Sarah E. Rowe, Lupeng Li, Laura E. Herring, Brian Conlon, Edward A. Miao, Blossom Damania, Anna Mae Diehl, and Pengda Liu

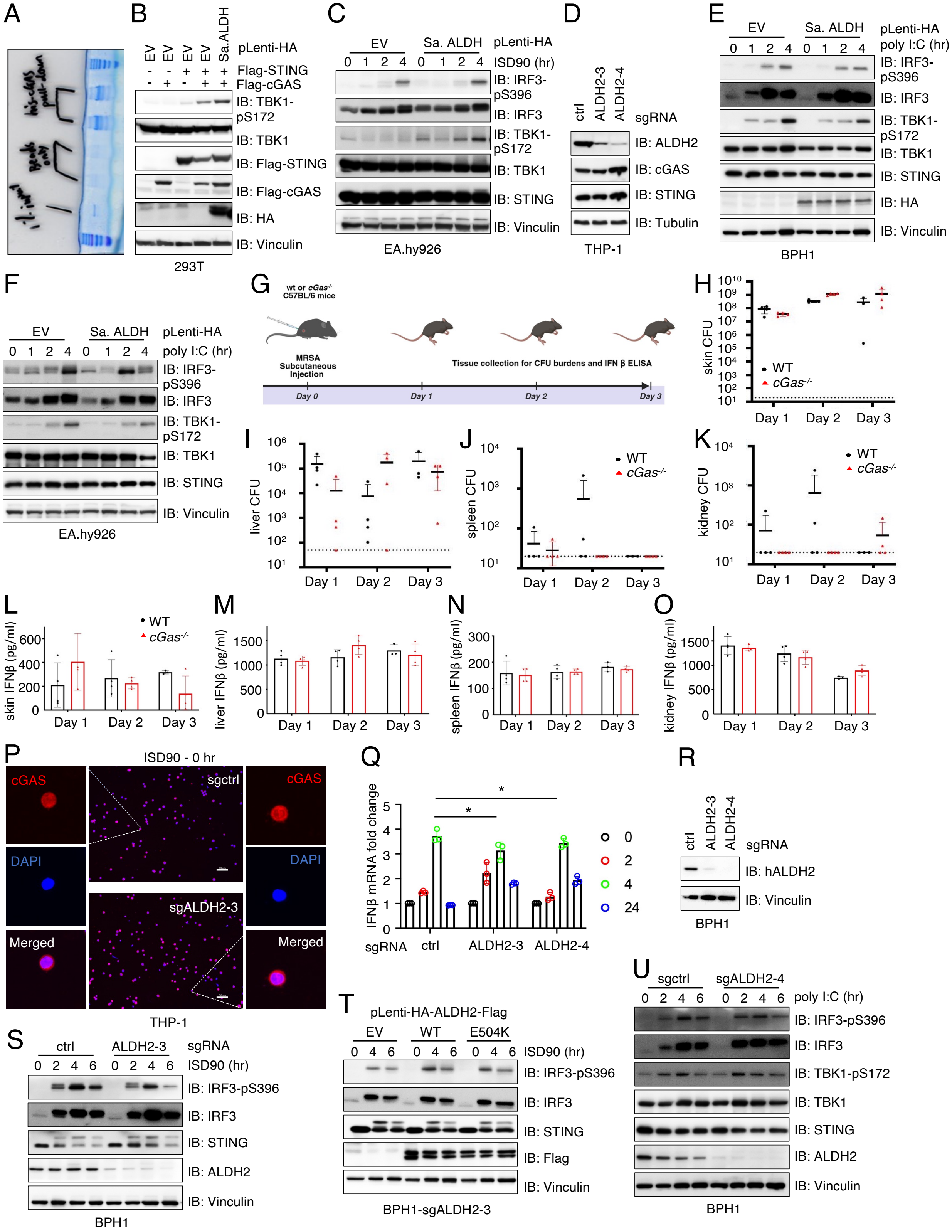
A
his-cGAS pull-down
Beads only
1:1 input
B
EV EV EV EV Sa.ALDH pLenti-HA
Flag-STING
Flag-cGAS
IB: TBK1-pS172
IB: TBK1
IB: Flag-STING
IB: Flag-cGAS
IB: HA
IB: Vinculin
293T
C
EV Sa. ALDH pLenti-HA
0 1 2 4 0 1 2 4 ISD90 (hr)
IB: IRF3-pS396
IB: IRF3
IB: TBK1-pS172
IB: TBK1
IB: STING
IB: Vinculin
EA.hy926
D
ctrl ALDH2-3 ALDH2-4 sgRNA
IB: ALDH2
IB: cGAS
IB: STING
IB: Tubulin
THP-1
E
EV Sa. ALDH pLenti-HA
0 1 2 4 0 1 2 4 poly I:C (hr)
IB: IRF3-pS396
IB: IRF3
IB: TBK1-pS172
IB: TBK1
IB: STING
IB: HA
IB: Vinculin
BPH1
F
EV Sa. ALDH pLenti-HA
0 1 2 4 0 1 2 4 poly I:C (hr)
IB: IRF3-pS396
IB: IRF3
IB: TBK1-pS172
IB: TBK1
IB: STING
IB: Vinculin
EA.hy926
G
wt or cGas-/- C57BL/6 mice
MRSA Subcutaneous Injection
Tissue collection for CFU burdens and IFN β ELISA
Day 0 Day 1 Day 2 Day 3
H
skin CFU
WT
cGas-/-
Day 1 Day 2 Day 3
I
liver CFU
Day 1 Day 2 Day 3
J
spleen CFU
WT
cGas-/-
Day 1 Day 2 Day 3
K
kidney CFU
WT
cGas-/-
Day 1 Day 2 Day 3
L
skin IFNβ (pg/ml)
WT
cGas-/-
Day 1 Day 2 Day 3
M
liver IFNβ (pg/ml)
Day 1 Day 2 Day 3
N
spleen IFNβ (pg/ml)
Day 1 Day 2 Day 3
O
kidney IFNβ (pg/ml)
Day 1 Day 2 Day 3
P
ISD90 - 0 hr
cGAS
DAPI
Merged
sgctrl
sgALDH2-3
THP-1
Q
IFNβ mRNA fold change
0 2 4 24
sgRNA ctrl ALDH2-3 ALDH2-4
R
ctrl ALDH2-3 ALDH2-4 sgRNA
IB: hALDH2
IB: Vinculin
BPH1
S
ctrl ALDH2-3 sgRNA
0 2 4 6 0 2 4 6 ISD90 (hr)
IB: IRF3-pS396
IB: IRF3
IB: STING
IB: ALDH2
IB: Vinculin
BPH1
T
pLenti-HA-ALDH2-Flag
EV WT E504K
0 4 6 0 4 6 0 4 6 ISD90 (hr)
IB: IRF3-pS396
IB: IRF3
IB: STING
IB: Flag
IB: Vinculin
BPH1-sgALDH2-3
U
sgctrl sgALDH2-4
0 2 4 6 0 2 4 6 poly I:C (hr)
IB: IRF3-pS396
IB: IRF3
IB: TBK1-pS172
IB: TBK1
IB: STING
IB: ALDH2
IB: Vinculin
BPH1

Figure S1. Sa.ALDH but not human ALDH2 facilitates cGAS activation.

(A) A representative Coomassie stained protein gel for his-cGAS protein pulldown products used for proteomics analysis to identify cGAS binding proteins using S. aureus cell lysates.

(B) IB analysis of WCL from HEK293T cells transfected with indicated DNA constructs.

(C) IB analysis of WCL from indicated EA.hy926 cells transfected with 5 ug/mL ISD90 for indicated time periods.

(D) IB analysis of indicated THP1 cells.

(E) IB analysis of WCL from indicated BPH1 cells transfected with 5 ug/mL poly I:C for indicated time periods.

(F) IB analysis of WCL from indicated EA.hy926 cells transfected with 5 ug/mL poly I:C for indicated time periods.

(G) A cartoon illustration of the RSA subcutaneous injection experiment procedure.

(H-K) Measurements of CFU from indicated organs at indicated time periods. Error bars were calculated as mean+/-SD, n=3 biological replicates. *$p<0.05$ (one-way ANOVA test).

(L-O) Representative IFNβ ELISA assay results using indicated organs at indicated time periods. Error bars were calculated as mean+/-SD, n=3 biological replicates. *$p<0.05$ (one-way ANOVA test).

(P) Representative IF images showing cGAS foci formation upon ISD90 stimulation in indicated THP1 cells.

(Q) RT-PCR analysis of IFNβ mRNA levels in indicated THP1 cells at indicated time periods post-ISD90 transfection.

(R) IB analysis of WCL from indicated BPH1 cells.

(S, T) IB analysis of WCL from indicated BPH1 cells transfected with 5 ug/mL ISD90 for indicated time periods.

(U) IB analysis of WCL from indicated BPH1 cells transfected with 5 ug/mL poly I:C for indicated time periods.

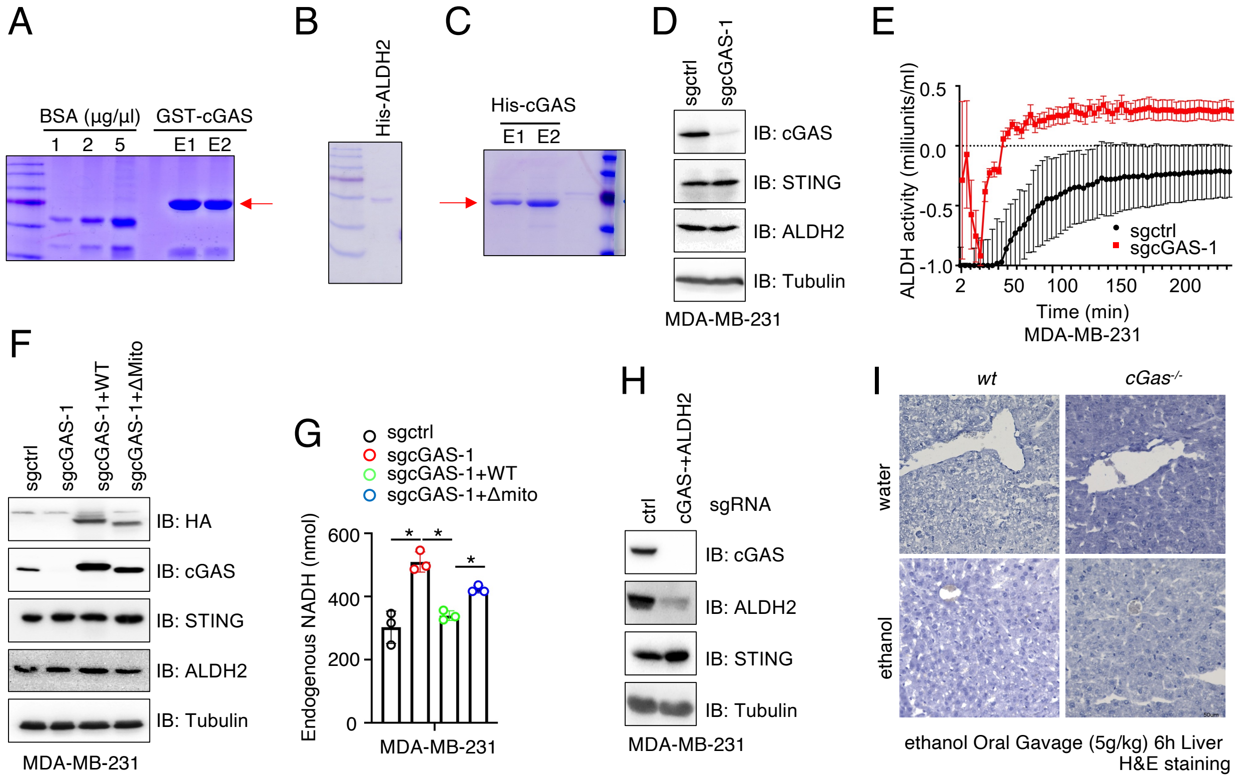

Figure S2. cGAS binds and suppresses ALDH2 activity.

(A-C) Representative Coomassie stained protein gels showing indicated bacterially purified cGAS and ALDH2 proteins.

(D, F, H) IB analysis of WCL from indicated MDA-MB-231 cells.

(E) Enzymatic activity of ALDH2 was measured in indicated MDA-MB-231 cells.

(G) Endogenous NADH levels were measured in indicated MDA-MB-231 cells. Error bars were calculated as mean+/-SD, n=3 biological replicates. *p<0.05 (one-way ANOVA test).

(I) Representative H&E staining of mouse livers from indicated animals 6 hrs post ethanol oral gavage.

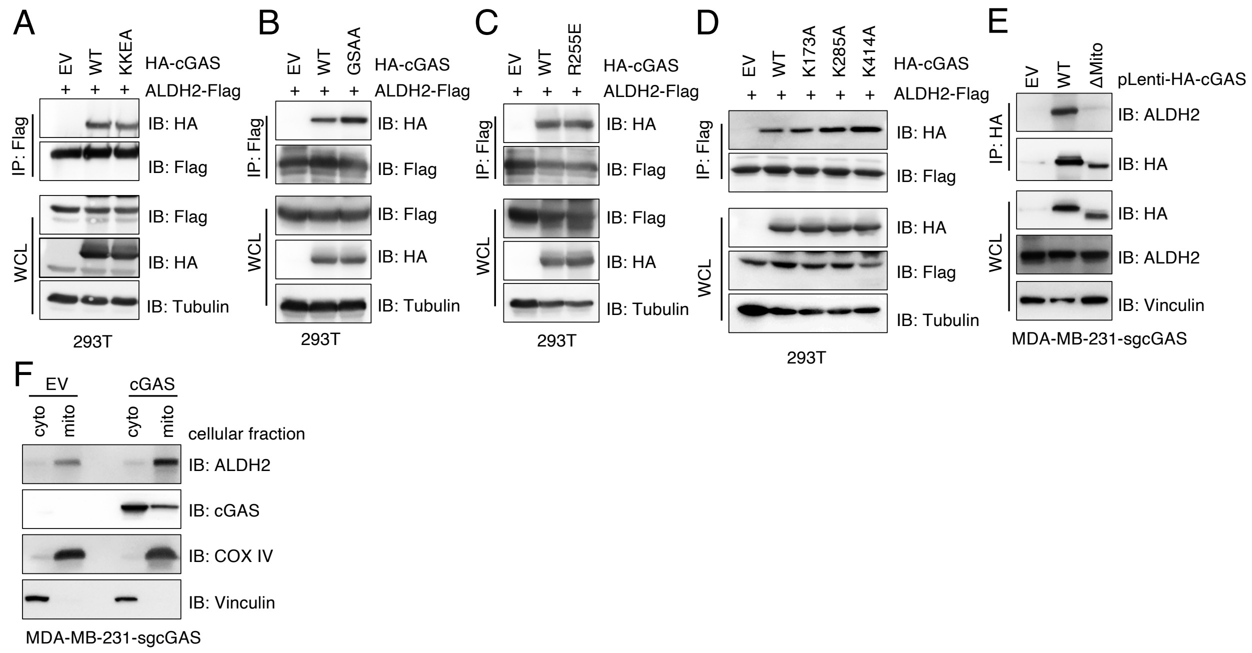

Figure S3. cGAS binding to ALDH2 is independent of its DNA binding and nucleosome tethering.

(A-D) IB analysis of Flag-IPs and WCL from HEK293T cells transfected with indicated ALDH2-Flag and cGAS constructs.

(E) IB analysis of HA-IPs and WCL from MDA-MB-231 cells depleted of endogenous cGAS reconstituted with WT or mito-localization signal-deleted cGAS.

(F) IB analysis of indicated cellular fractions from MDA-MB-231 cells depleted of endogenous cGAS reconstituted with WT-cGAS or EV.

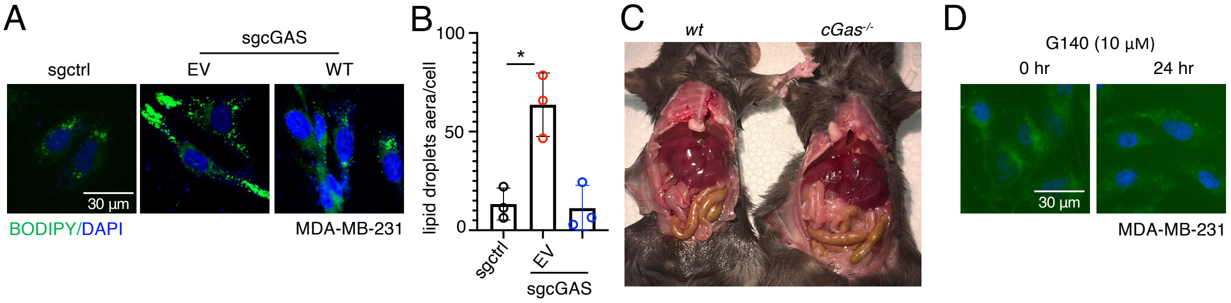

Figure S4. Genetic cGAS depletion increases cellular lipid droplets.
(A) Representative IF images for lipid droplets staining by BODIPY in indicated MDA-MB-231 cells and quantified in (B). Error bars were calculated as mean+/-SD, n=3 biological replicates. *p<0.05 (one-way ANOVA test).
(C) Representative images for livers from *wt* or *cGas*$^{-/-}$ C57BL/6 mice.
(D) Representative IF images for lipid droplets staining by BODIPY in indicated MDA-MB-231 cells treated with 10 uM G140 for indicated time periods.

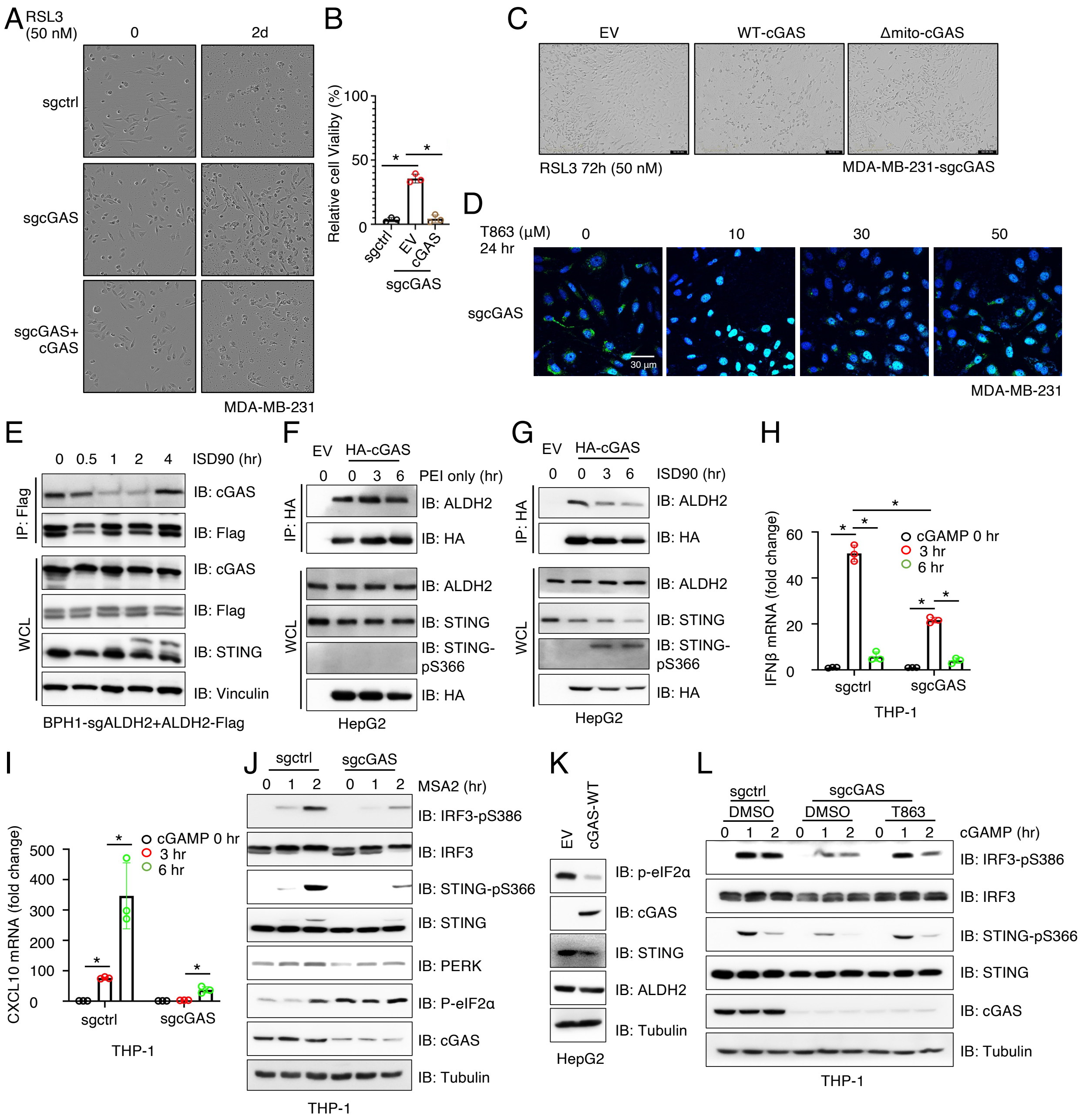

Figure S5. cGAS depletion causes ferroptosis resistance and dampened STING signaling due to lipid droplet accumulation.

(A) RSL3 at 50 nM concentration was used to induce ferroptosis in indicated MDA-MB-231 cells. Images were captured using IncuCyte® Live-Cell Analysis equipment and relative cell viability was quantified in (B).

(C) RSL3 at 50 nM concentration was used to induce ferroptosis in indicated MDA-MB-231 cells for 72 hrs.

(D) Representative IF images for lipid droplets staining by BODIPY in indicated MDA-MB-231 cells. Where indicated, indicated doses of T863 was used to treat MDA-MB-231-sgcGAS cells for 24 hrs.

(E) IB analysis of Flag-IPs and WCL from indicated BPH1 cells transfected with 5 ug/mL ISD90 for indicated time periods.

(F, G) IB analysis of HA-IPs and WCL from indicated HepG2 cells transfected with PEI only (F) or 5 ug/mL ISD90 (G) for indicated time periods.

(H, I) RT-PCR analysis of IFNβ (H) or CXCL10 (I) mRNA levels in control or cGAS-depleted THP1 treated with 2.5 ug/mL 2'3'-cGAMP for indicated time periods. Error bars were calculated as mean+/-SD, n=3 biological replicates. *$p<0.05$ (one-way ANOVA test).

(J) IB analysis of WCL from indicated THP1 cells treated with 20 μM MSA2 for indicated time periods.

(K) IB analysis of WCL from indicated HepG2 cells.

(L) IB analysis of WCL from indicated THP1 cells treated with 2.5 ug/mL 2'3'-cGAMP for indicated time periods. Where indicated, 30 μM T863 was pre-incubated with cells prior to 2'3'-cGAMP addition.

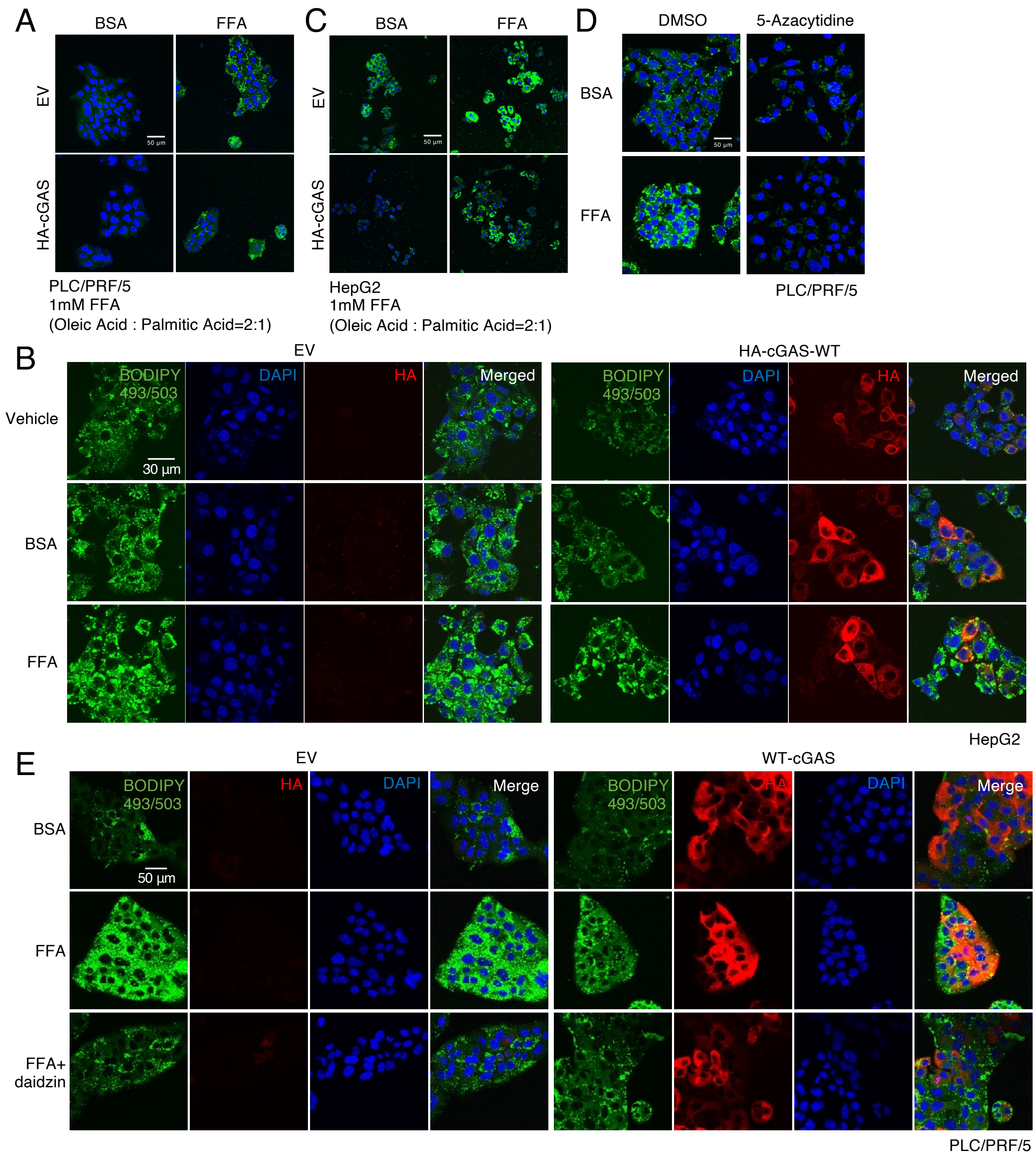

Figure S6. cGAS depletion facilitates FFA-induced lipid droplet accumulation in liver cells.

(A, C) Representative IF images for lipid droplets staining by BODIPY in control or cGAS-WT stably expressing PLC (A) or HepG2 (C) cells treated with FFA or BSA control.

(B) Representative IF images for lipid droplets staining by BODIPY in control or cGAS-WT stably expressing HepG2 cells treated with FFA or BSA control.

(D) Representative IF images for lipid droplets staining by BODIPY in PLC cells treated with FFA or BSA control. Where indicated, cells were treated with 2 μM 5-Azacytine for 3 days prior to FFA treatment.
(E) Representative IF images for lipid droplets staining by BODIPY in control or cGAS-WT stably expressing PLC cells treated with FFA stimulation with or without daidzin treatment.

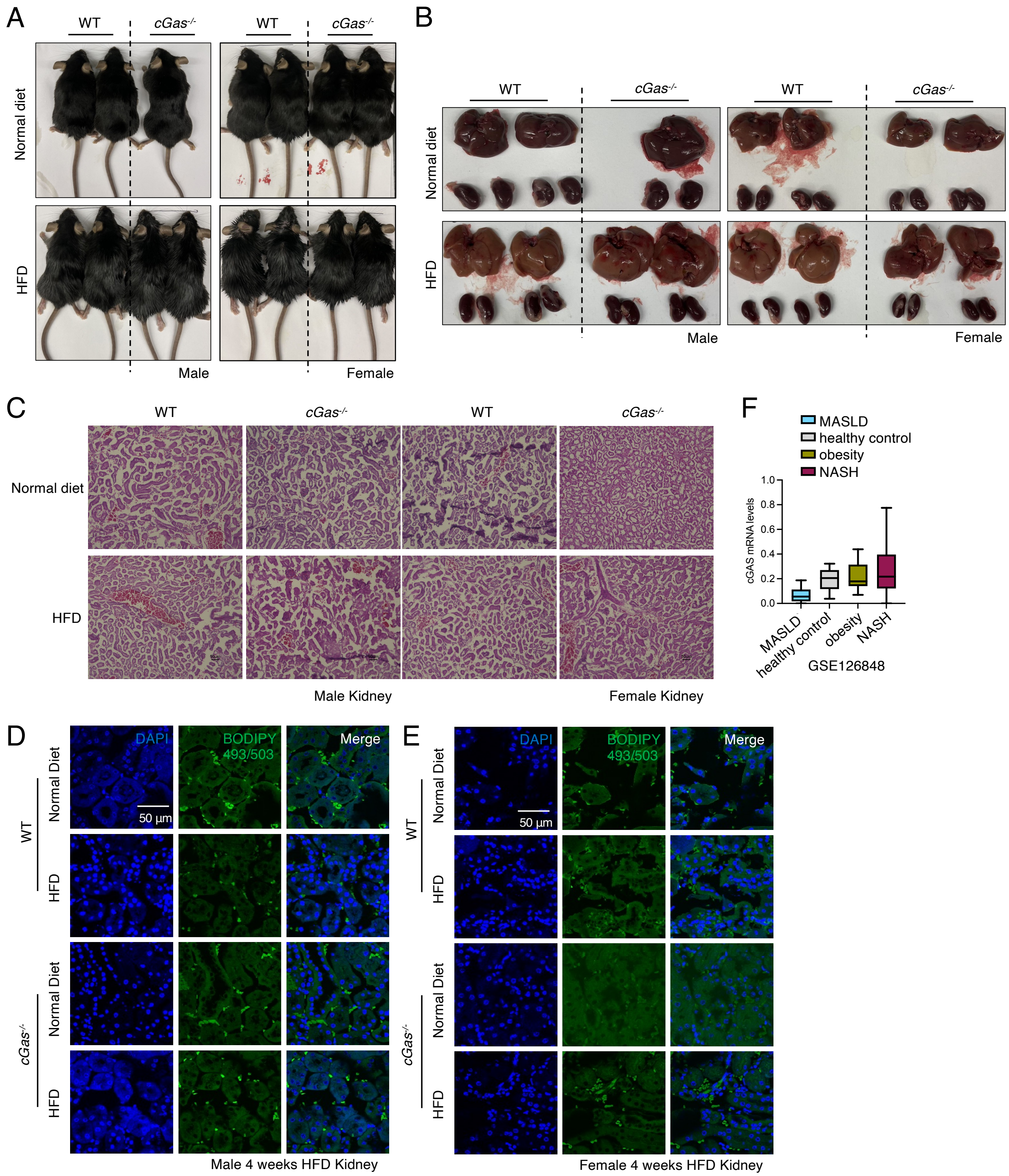

Figure S7. *cGas*$^{-/-}$ mice develop more server MASLD upon HFD.

(A) Representative images for indicated animal receiving indicated diets at the end of the experiments.

(B) Representative images for collected organs from indicated animals received indicated diets.

(C) Representative H&E staining for kidney slides from indicated animals received indicated diets.
(D) Representative IF images for lipid droplets staining by BODIPY in indicated animal kidneys received indicated diets.

Supplemental Methods

1. Cloning primers used in this study

hALDH2-BamHI-F: GCATGGATCC TTGCGCGCTGCCGC
hALDH2-SalI-R: GCATGTCGACTTATGAGTTCTTCTGAGGCACTTTGA
hALDH2-sg3-F: CACCGCCCACCGTCAATCCGTCCAC
hALDH2-sg3-R: AAACGTGGACGGATTGACGGTGGGC
hALDH2-sg4-F: CACCGCTACACACGCCATGAACCTG
hALDH2-sg4-R: AAACCAGGTTCATGGCGTGTGTAGC
SaALDH-SalI-F: GCATGTCGACGCAGTAAACGTTCGAGATTATATTGC
SaALDH-BamHI-R: GCATGGATCCTTAGTACAAACCTTTTAAAGCATTGCTTGTATC
Saffh-SalI-F: GCATGTCGACGCATTTGAAGGGTTATCAGAACGCTTG
Saffh-BamHI-R: GCATGGATCCTTAAAACGGTAAATTCATACCTTTTAAC
SaGTF-SalI-F: GCATGTCGACAAATTTTCAGTAATAGTTCCAACATACAATTCAG
SaGTF-BamHI-R: GCATGGATCCTTATTTATTTAGTGGATAAGTGATATGTG
SaCshB-BamHI-F: GCATGGATCCGCAAAACATCCATTCGAACAATTTAATCTAG
SaCshB-SalI-R: GCATGTCGACCTAACCTTTTTTGTTTTGCTTACGTTTTTGTC
cGAS-BglII-F: GCATAGATCTCAGCCTTGGCACGGAAAGGC
cGAS-XhoI-R: GCATCTCGAGTCAAAATTCATCAAAAACTGG
cGAS-sgRNA-F: CACCGGCACGTGCTCATAGTAGCTCC
cGAS-sgRNA-R: AAACGGAGCTACTATGAGCACGTGCC
cGAS-deltaMTS (161-190)-F: GGGATGCGGCGCCTGACCACCTGCTGCTC
cGAS-deltaMTS (161-190)-R: GAGCAGCAGGTGGTCAGGCGCCGCATCCC
CGAS-K414A-F: GATTGTTTAAAACTAATGGCATACCTTTTAGAACAGCTGAAAG
CGAS-K414A-R: CTTTCAGCTGTTCTAAAAGGTATGCCATTAGTTTTAAACAATC
CGAS-K285A-F: GTTTAGGAAAATCATTGCGGAAGAAATTAACGACATTAAAGATAC
CGAS-K285A-R: GTATCTTTAATGTCGTTAATTTCTTCCGCAATGATTTTCCTAAAC
CGAS-K173A-F: GGTTTTGGAGAAGTTGGCGCTCAGCCGCGATG
CGAS-K173A-R: CATCGCGGCTGAGCGCCAACTTCTCCAAAACC
CGAS-G212AS213A-F: CGGGCTGCTGAACACCGCCGCCTACTATGAGCACGTGAAG
CGAS-G212AS213A-R: CTTCACGTGCTCATAGTAGGCGGCGGTGTTCAGCAGCCCG
cGAS-K407EK411A-F:
GAGAAATGTTGCAGGGAGGATTGTTTAGCGCTAATGAAATACCTTTTAGAACAGC
cGAS-K407EK411A-R:
GCTGTTCTAAAAGGTATTTCATTAGCGCTAAACAATCCTCCCTGCAACATTTCTC

2. RT-PCR primers used in this study

hIFN β-F: 5'-TCTCCTCCAAATTGCTCTCC-3'
hIFN β-R: 5'-CTCCCATTCAATTGCCACAG-3'
PLIN1-F: 5'-TGGGTGGTGTGGCACATAC-3'
PLIN1-R: 5'-CCTCCCCTTGGTTGAGGAGA-3'
PLIN2-F: 5'-TTGCAGTTGCCAATACCTATGC-3'
PLIN2-R: 5'-CCAGTCACAGTAGTCGTCACA-3'
PLIN3-F: 5'-GCCCAAGAGATGGTGTCTAGC-3'
PLIN3-R: 5'-CCGGTCACTACGGACTTTGT-3'
PLIN4-F: 5'-GGAGCTGCAACCTTCGGAAA-3'
PLIN4-R: 5'-GGACCACTCCCTTAGCCAC-3'

PLIN5-F: 5’-AAGGCCCTGAAGTGGGTTC-3’
PLIN5-R: 5’-GCATGTGGTCTATCAGCTCCA-3’
SREBP1-F: 5’-ACAGTGACTTCCCTGGCCTAT-3’
SREBP1-R: 5’-CATGGACGGGTACATCTTCAA-3’
FSP27-F: 5’-ATTGATGTGGCCCGTGTAACG-3’
FSP27-R: 5’-CAGCAGTGCAGATCATAGGAAA-3’
ATGL-F: 5’-ATGGTGGCATTTCAGACAACC-3’
ATGL-R: 5’-CGGACAGATGTCACTCTCGC-3’

3. Key resources table

REAGENT or RESOURCE	SOURCE	IDENTIFIER
**Beads and Recombinant Proteins**		
Glutathione agarose beads	GE Healthcare	cat#17-0756-05
Nickel HTC Agarose beads	GoldBio	cat#R-202-100
Anti-HA agarose beads	Sigma-Aldrich	cat#A-2095
Anti-Flag agarose beads	Sigma-Aldrich	cat#A2220
**Chemicals/Compounds**		
Protease Inhibitor Cocktail	Apexbio Technology	cat#K1008
Phosphatase Inhibitor Cocktail	Apexbio Technology	cat#K1015
DMEM	Gibco	Cat# 2786121
RPMI Medium 1640	Gibco	Cat# 3102104
Daidzin	Sigma Aldrich	Cat# 30408
T863	Sigma Aldrich	Cat# SML0539
Puromycin dihydrochloride	Sigma Aldrich	Cat# P8833
Blasticidin S hydrochloride	Sigma Aldrich	Cat# 15205
2’3’-cGAMP	InvivoGen	Cat# tlrl-nacga23-02
Protease Inhibitor Cocktail	Bimake	Cat# B14012
Phosphatase inhibitor cocktail A and B	Bimake	Cat# B15001-A /B15001-B
RSL3	MedChemExpress	Cat# HY-100218A
Palmitic Acid	The Lab Depot	Cat# P1513-500GM
Oleic Acid	Sigma Aldrich	Cat# O7501
ISD90	IDT	5’- TACAGATCTACTAGTGATCTATGACTGATCTGTACATGATCTACA -3’
Poly I:C	IDT	5’-TACAGATCTACTAGTGATCTATGACTGATCTGTACATGATCTACATACAGATCTACTAGTGATCTATGACTGATCTGTACATGATCTACA-3’
**Antibodies**		
Anti-p-IRF-3 (Ser386) antibody	Cell Signaling Technology	cat#37829
Anti-p-IRF-3 (Ser396) antibody	Cell Signaling Technology	cat#29047
Anti-p-STING (Ser366)	Cell Signaling Technology	cat#50907

Anti-p-TBK1 (Ser172) antibody	Cell Signaling Technology	cat#5483
Anti-STING antibody	Cell Signaling Technology	cat#13647
Anti-rabbit IgG, HRP-linked antibody	Cell Signaling Technology	cat#7074
Anti-mouse IgG, HRP-linked antibody	Cell Signaling Technology	cat#7076
Anti-GST antibody	Santa Cruz Biotechnology	cat#sc-459
Anti-vinculin antibody	Santa Cruz Biotechnology	cat#sc-25336
Anti-Flag antibody	Sigma-Aldrich	cat#F1825
Anti-Flag antibody	Sigma-Aldrich	cat#F7425
Anti-Tubulin antibody	Sigma-Aldrich	cat#T5168
Anti-HA antibody	Proteintech	cat#51064-2-AP
Anti-His-Tag antibody	Proteintech	cat#66005-1-Ig
IRF-3 Mouse mAb	Cell Signaling Technology	Cat# 10949
ALDH2 Rabbit Antibody	Cell Signaling Technology	Cat# 18818
Phospho-eIF2α (Ser51) Rabbit mAb	Cell Signaling Technology	Cat# 3398
Phospho-TBK1/NAK (Ser172) Rabbit mAb	Cell Signaling Technology	Cat# 5483
TBK1/NAK Rabbit Antibody	Cell Signaling Technology	Cat# 3013
HA-Tag Rabbit mAb	Cell Signaling Technology	Cat# 3724
6*His, His-Tag Monoclonal mouse antibody	Proteintech	Cat# 66005-1-Ig
Monoclonal anti-Tubulin antibody	Sigma Aldrich	Cat# T-5168
Anti-Vinculin antibody	Santa Cruz Biotechnology	Cat# sc-25336
Goat anti-Mouse IgG (H+L) Secondary Antibody, Alexa Fluor™ 488	Invitrogen	Cat# A-11001
Goat anti-Rabbit IgG (H+L) Secondary Antibody, Alexa Fluor™ 488	Invitrogen	Cat# A-11008
Goat anti-Mouse IgG (H+L) Secondary Antibody, Alexa Fluor™ 594	Invitrogen	Cat# A-11005
Goat anti-Rabbit IgG (H+L) Secondary Antibody, Alexa Fluor™ 594	Invitrogen	Cat# A-11012
Goat anti-Rabbit IgG (H+L) Highly Secondary Antibody, Alexa Fluor™ 647	Invitrogen	Cat# A-21245
**Transfection Reagents and Antibiotics**		
Lipofectamine 3000	Thermo Fisher Scientific	cat# L3000150
Polyethylenimine (PEI)	Polysciences, Inc.	cat# 23866-1
Puromycin	Fisher BioReagents	cat# 58-58-2
Hygromycin	Sigma-Aldrich	cat# H3274

**Software**		
Graphpad Prism 8	Prism	
**Others**		
RNA miniprep super kit	BioBasic	cat#BS584
iScript™ Reverse Transcription Supermix for RT-qPCR	Bio-Rad	cat#170-8891
iTaq universal SYBR green supermix	Bio-Rad	cat#172-5124
ProLong™ Gold Antifade Mountant with DAPI	Invitrogen	cat# P36931
Protein A/G XPure Agarose Resin	UBPBio	cat#P5030-5
QuickExtract DNA Extraction Solution	Bioresearch technologies	cat#QE09050
ELISA kit	R&D systems	Cat# DY814-05, lot#P181016
Protein Bradford Assay reagent	Biorad	Cat# 5000006
BSA	Goldbio	Cat# A-420-500
3xSDS sample buffer	home-made	6.7% SDS, 33.3% glycerol, 300 mM DTT and a bit BPB
PVDF membrane	Biorad	Cat# 1620177
ECL-millipore	Millipore	Cat# WBKLS0500
ECL-Femto	Thermo Scientific	Cat# 34096
ECL-Pirece	Thermo Scientific	Cat# 32106
