# Supplementary material for: cGAS Inhibits ALDH2 to Suppress Lipid Droplet Function and Regulate MASLD Progression
Source: Adv Sci (Weinh). 2025 Oct 3;12(46):e08576. doi: 10.1002/advs.202508576 (PMC12697864; doi:10.1002/advs.202508576)
Supplement: Supplementary file 2 — Supporting Information [file ADVS-12-e08576-s002.pptx]

## Slide 1
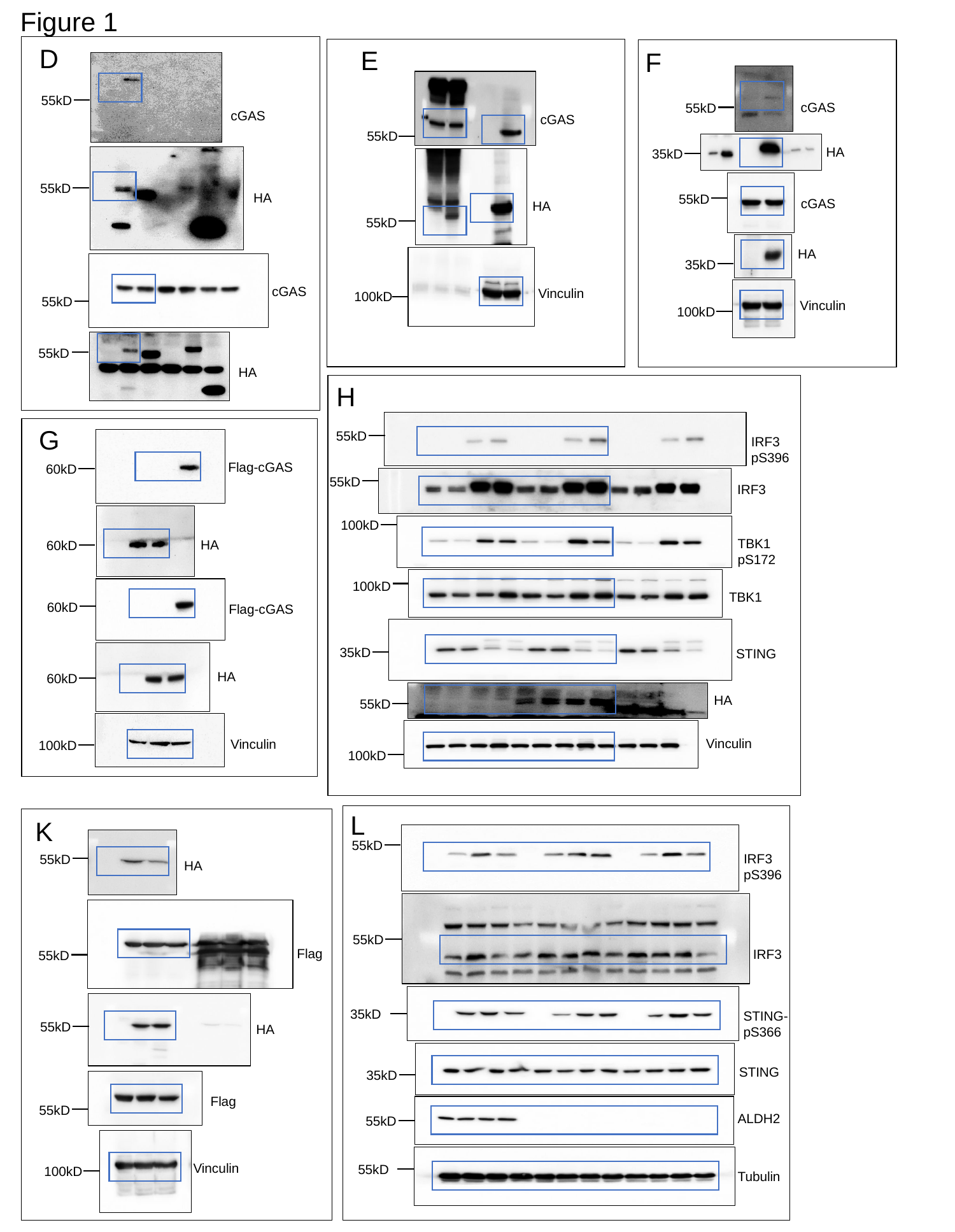

Figure 1
D
55kD
cGAS
55kD
HA
cGAS
55kD
55kD
HA
E
cGAS
55kD
HA
55kD
Vinculin
100kD
F
cGAS
55kD
HA
35kD
55kD
cGAS
HA
35kD
Vinculin
100kD
H
55kD
55kD
100kD
100kD
35kD
HA
55kD
Vinculin
100kD
IRF3
pS396
IRF3
TBK1
pS172
TBK1
STING
G
Flag-cGAS
60kD
HA
60kD
60kD
Flag-cGAS
HA
60kD
Vinculin
100kD
L
55kD
55kD
35kD
STING-pS366
STING
35kD
ALDH2
55kD
55kD
Tubulin
IRF3
pS396
IRF3
K
55kD
HA
55kD
55kD
HA
Flag
55kD
Vinculin
100kD
Flag

## Slide 2
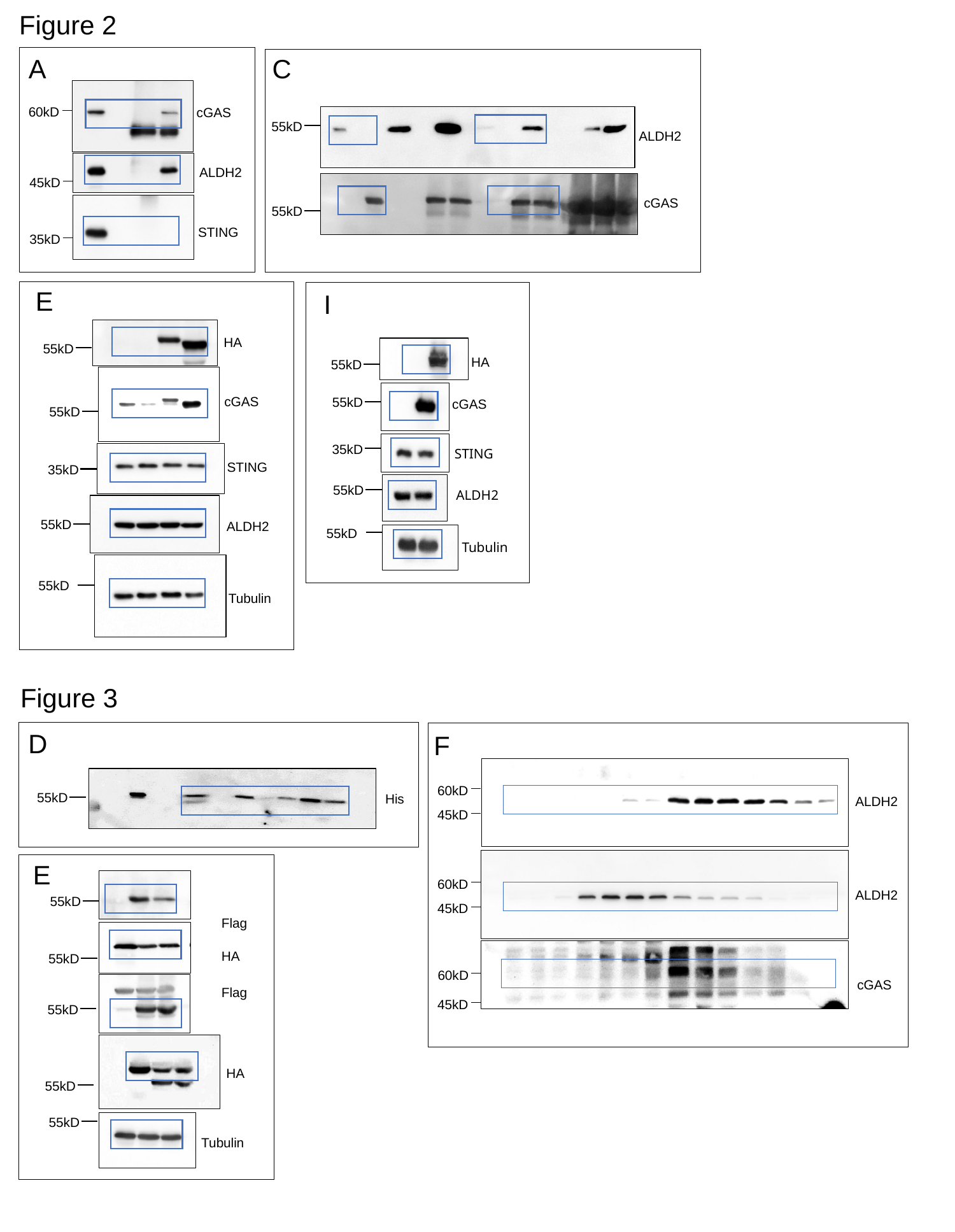

Figure 2
A
60kD
cGAS
ALDH2
45kD
35kD
C
55kD
ALDH2
cGAS
55kD
STING
E
I
HA
cGAS
STING
ALDH2
Tubulin
55kD
55kD
35kD
55kD
55kD
HA
55kD
cGAS
55kD
STING
35kD
55kD
ALDH2
55kD
Tubulin
Figure 3
D
55kD
His
F
60kD
ALDH2
45kD
60kD
ALDH2
45kD
60kD
cGAS
45kD
E
55kD
Flag
HA
55kD
Flag
55kD
HA
55kD
55kD
Tubulin

## Slide 3
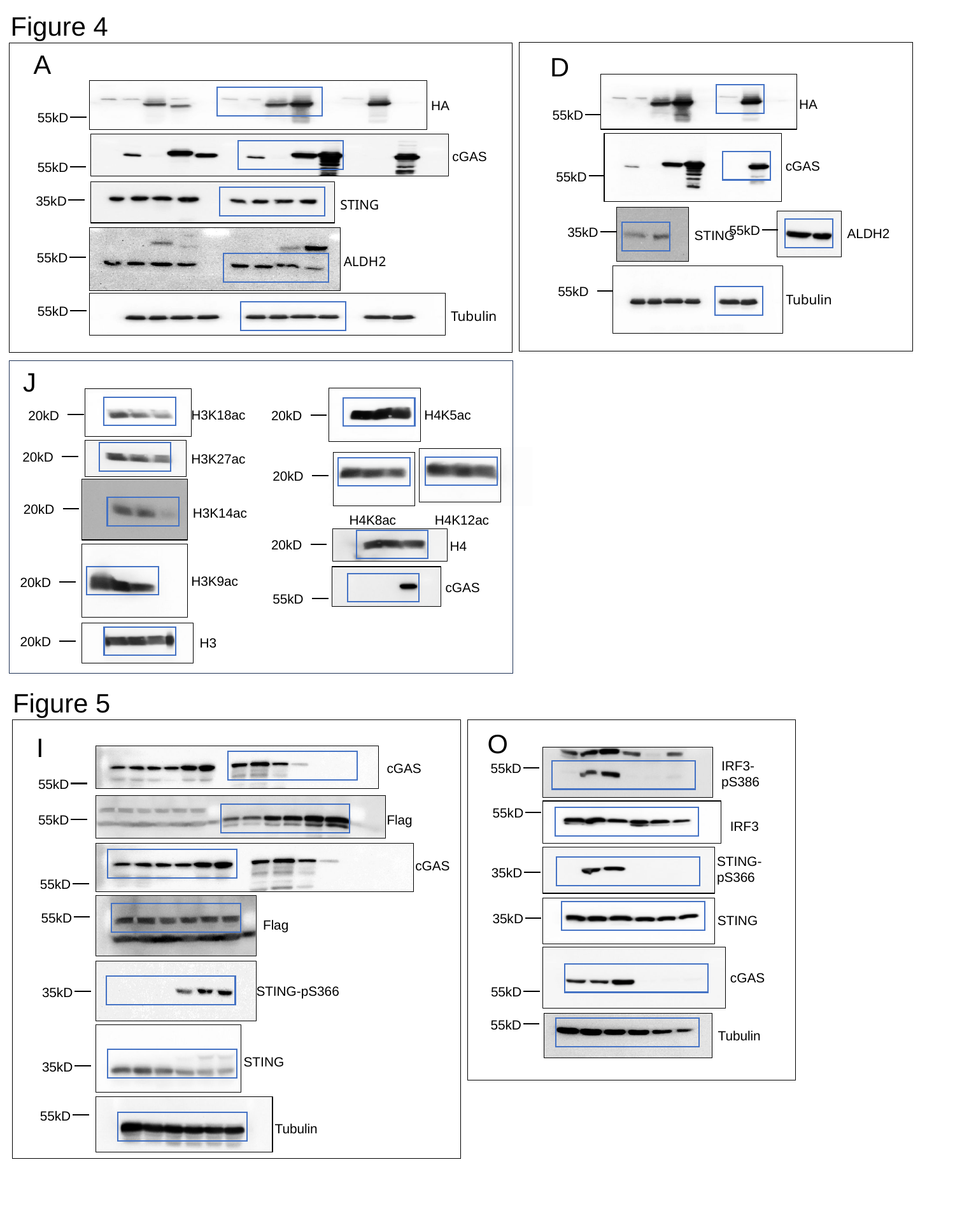

Figure 4
A
HA
55kD
cGAS
55kD
35kD
STING
55kD
ALDH2
55kD
Tubulin
D
HA
55kD
cGAS
55kD
55kD
35kD
ALDH2
STING
55kD
Tubulin
J
20kD
20kD
H3K18ac
H4K5ac
20kD
H3K27ac
20kD
20kD
H3K14ac
H4K8ac
H4K12ac
20kD
H4
H3K9ac
20kD
cGAS
55kD
20kD
H3
Figure 5
O
IRF3-pS386
55kD
55kD
IRF3
STING-pS366
35kD
35kD
STING
cGAS
55kD
55kD
Tubulin
I
cGAS
55kD
55kD
Flag
cGAS
55kD
55kD
Flag
35kD
STING-pS366
STING
35kD
55kD
Tubulin

## Slide 4
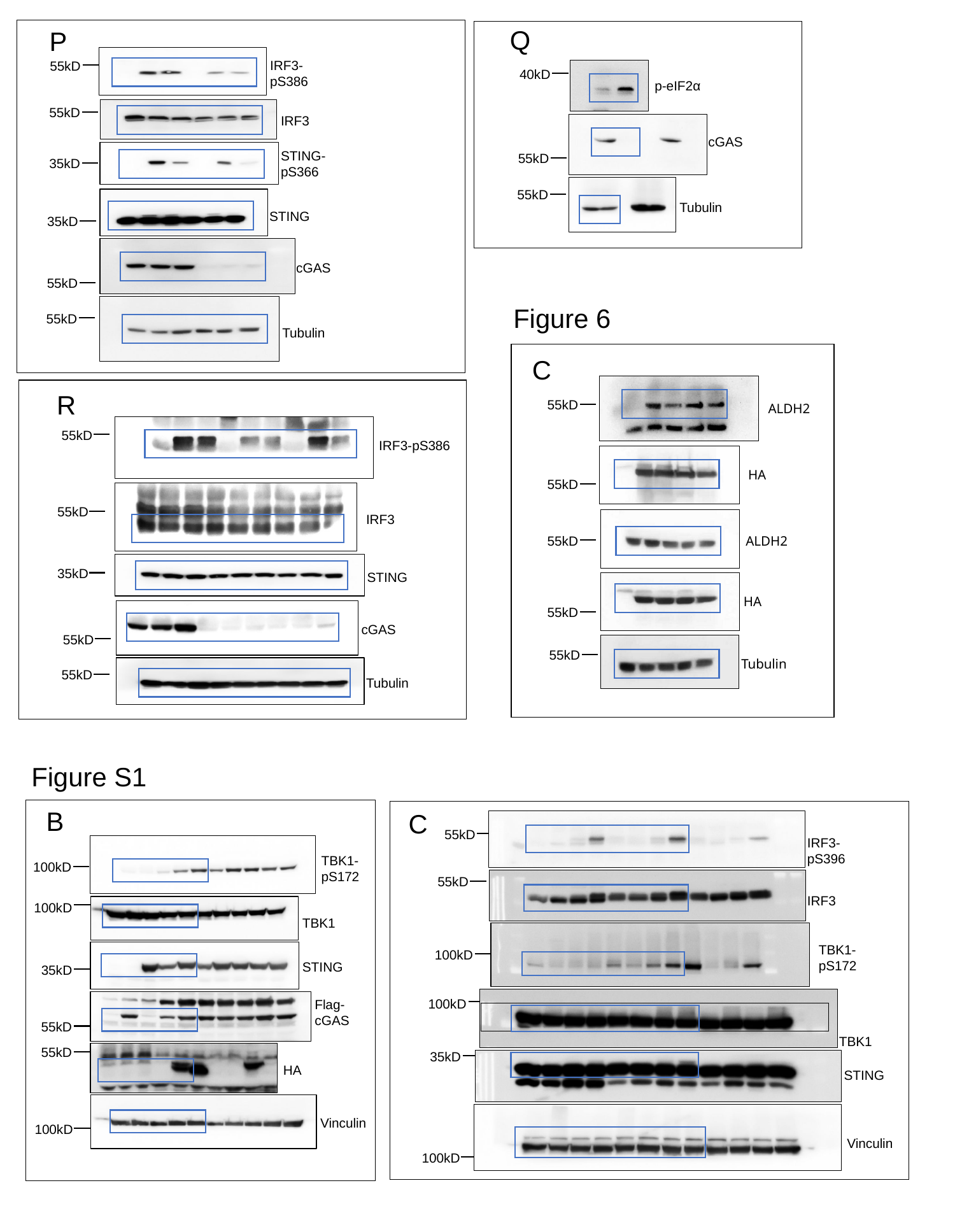

Q
40kD
p-eIF2α
cGAS
55kD
55kD
Tubulin
P
55kD
IRF3-pS386
55kD
IRF3
STING-pS366
35kD
STING
35kD
cGAS
55kD
55kD
Tubulin
Figure 6
C
55kD
ALDH2
HA
55kD
55kD
ALDH2
HA
55kD
55kD
Tubulin
R
55kD
IRF3-pS386
55kD
IRF3
35kD
STING
cGAS
55kD
55kD
Tubulin
Figure S1
B
TBK1-pS172
100kD
100kD
TBK1
STING
35kD
Flag-cGAS
55kD
55kD
HA
Vinculin
100kD
C
55kD
IRF3-pS396
55kD
IRF3
TBK1-pS172
100kD
100kD
TBK1
35kD
STING
Vinculin
100kD

## Slide 5
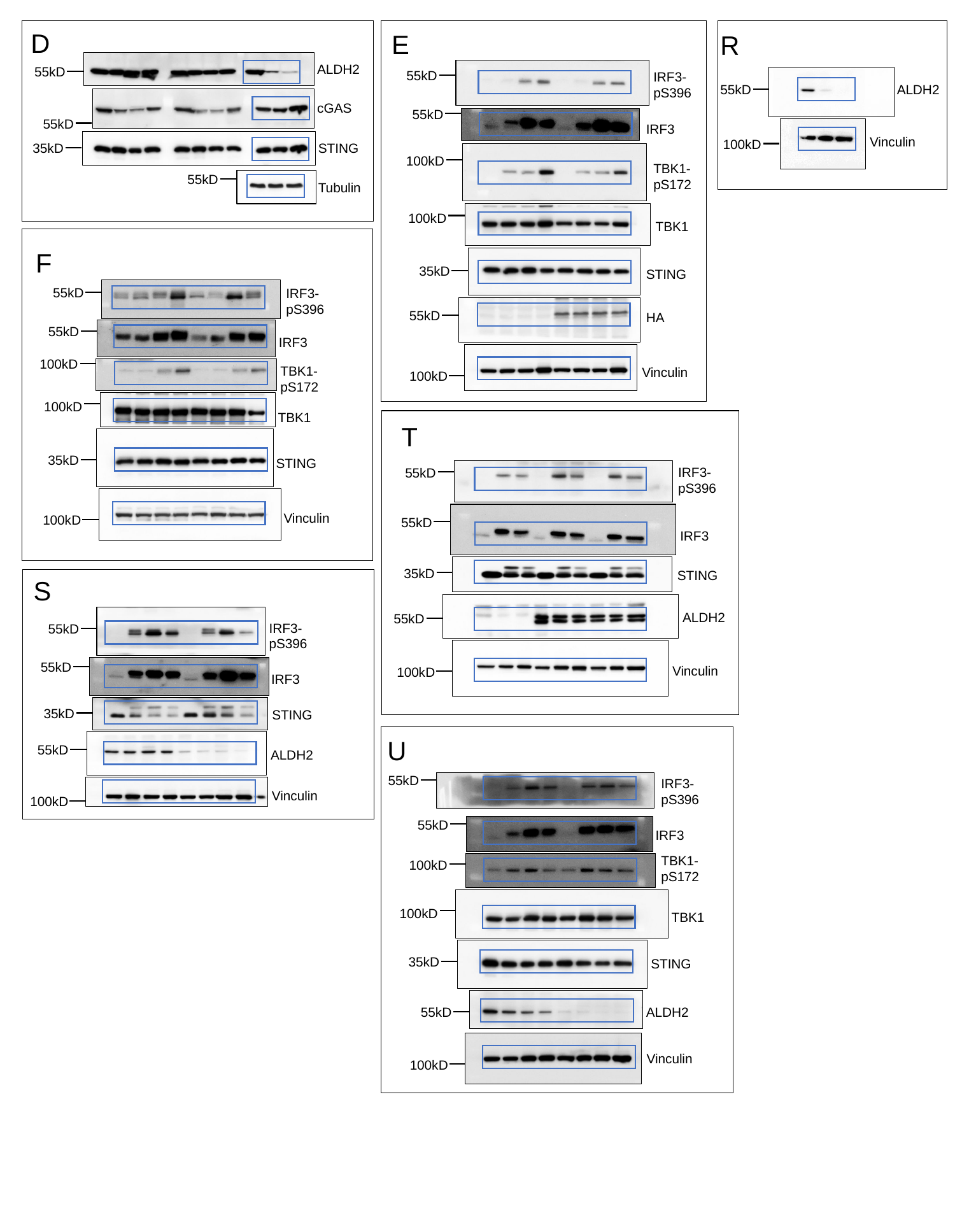

D
ALDH2
55kD
cGAS
55kD
35kD
STING
55kD
Tubulin
E
55kD
IRF3-
pS396
55kD
IRF3
100kD
TBK1-
pS172
100kD
TBK1
35kD
STING
55kD
HA
Vinculin
100kD
R
55kD
ALDH2
Vinculin
100kD
F
55kD
IRF3-
pS396
55kD
IRF3
100kD
TBK1-
pS172
100kD
TBK1
35kD
STING
Vinculin
100kD
T
55kD
IRF3-pS396
55kD
IRF3
35kD
STING
ALDH2
55kD
Vinculin
100kD
S
55kD
IRF3-pS396
55kD
IRF3
35kD
STING
55kD
ALDH2
Vinculin
100kD
U
55kD
IRF3-pS396
55kD
IRF3
TBK1-pS172
100kD
100kD
TBK1
35kD
STING
55kD
ALDH2
Vinculin
100kD

## Slide 6
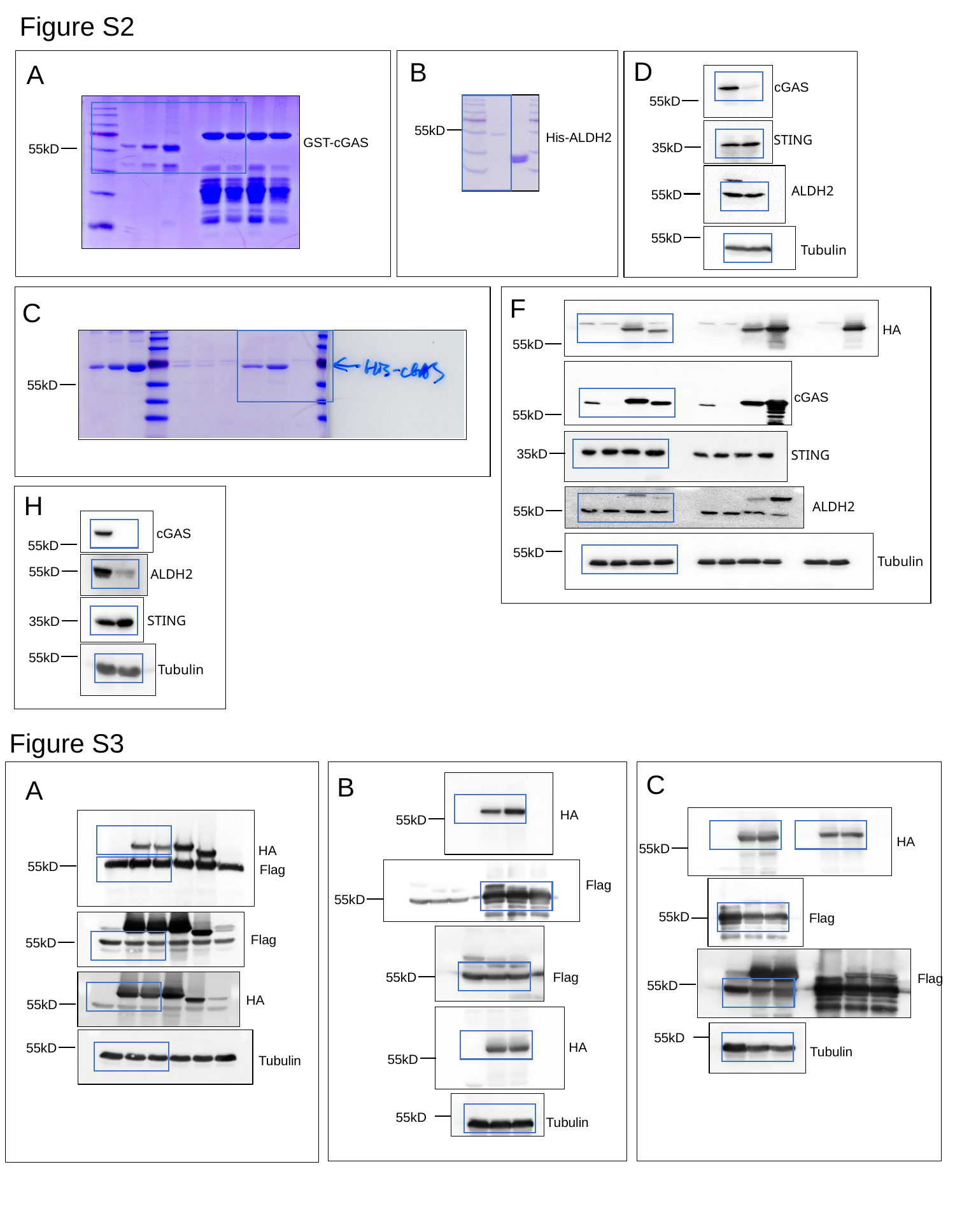

Figure S2
D
 cGAS
55kD
STING
35kD
ALDH2
55kD
55kD
Tubulin
B
His-ALDH2
55kD
A
GST-cGAS
55kD
F
HA
55kD
cGAS
55kD
35kD
STING
ALDH2
55kD
55kD
Tubulin
C
55kD
H
55kD
55kD
35kD
55kD
cGAS
ALDH2
STING
Tubulin
Figure S3
A
HA
55kD
Flag
Flag
55kD
HA
55kD
55kD
Tubulin
C
HA
55kD
55kD
Flag
Flag
55kD
55kD
Tubulin
B
HA
55kD
55kD
55kD
Flag
HA
55kD
55kD
Flag
Tubulin

## Slide 7
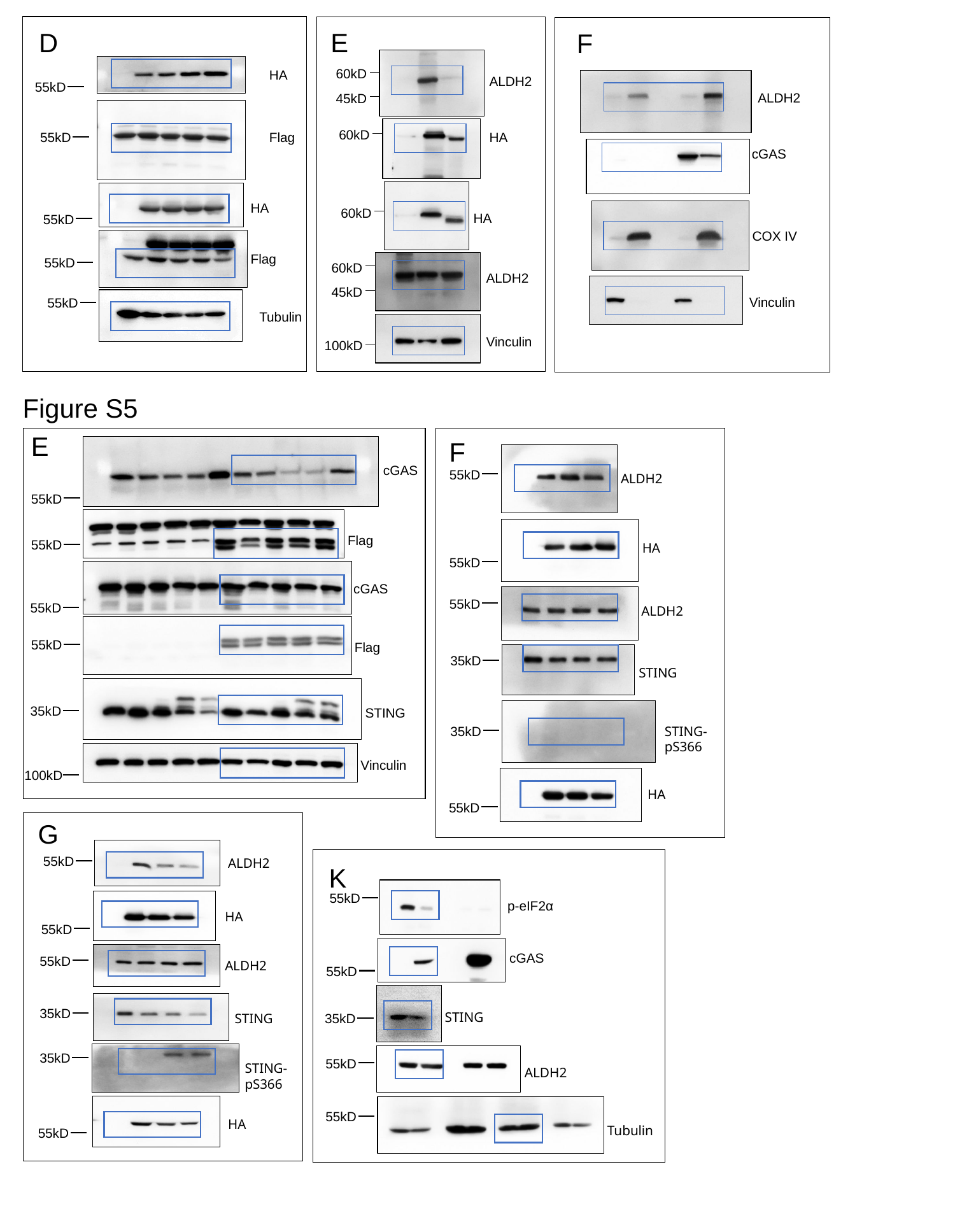

D
55kD
55kD
HA
55kD
Flag
55kD
55kD
E
60kD
ALDH2
45kD
60kD
HA
60kD
HA
60kD
ALDH2
45kD
Vinculin
100kD
F
HA
ALDH2
cGAS
COX IV
Vinculin
Flag
Tubulin
Figure S5
E
cGAS
55kD
Flag
55kD
cGAS
55kD
55kD
Flag
35kD
STING
Vinculin
100kD
F
55kD
ALDH2
HA
55kD
55kD
ALDH2
35kD
STING
35kD
STING-pS366
HA
55kD
G
55kD
ALDH2
HA
55kD
55kD
ALDH2
35kD
STING
35kD
STING-pS366
HA
55kD
K
55kD
p-eIF2α
cGAS
55kD
35kD
STING
55kD
ALDH2
55kD
Tubulin

## Slide 8
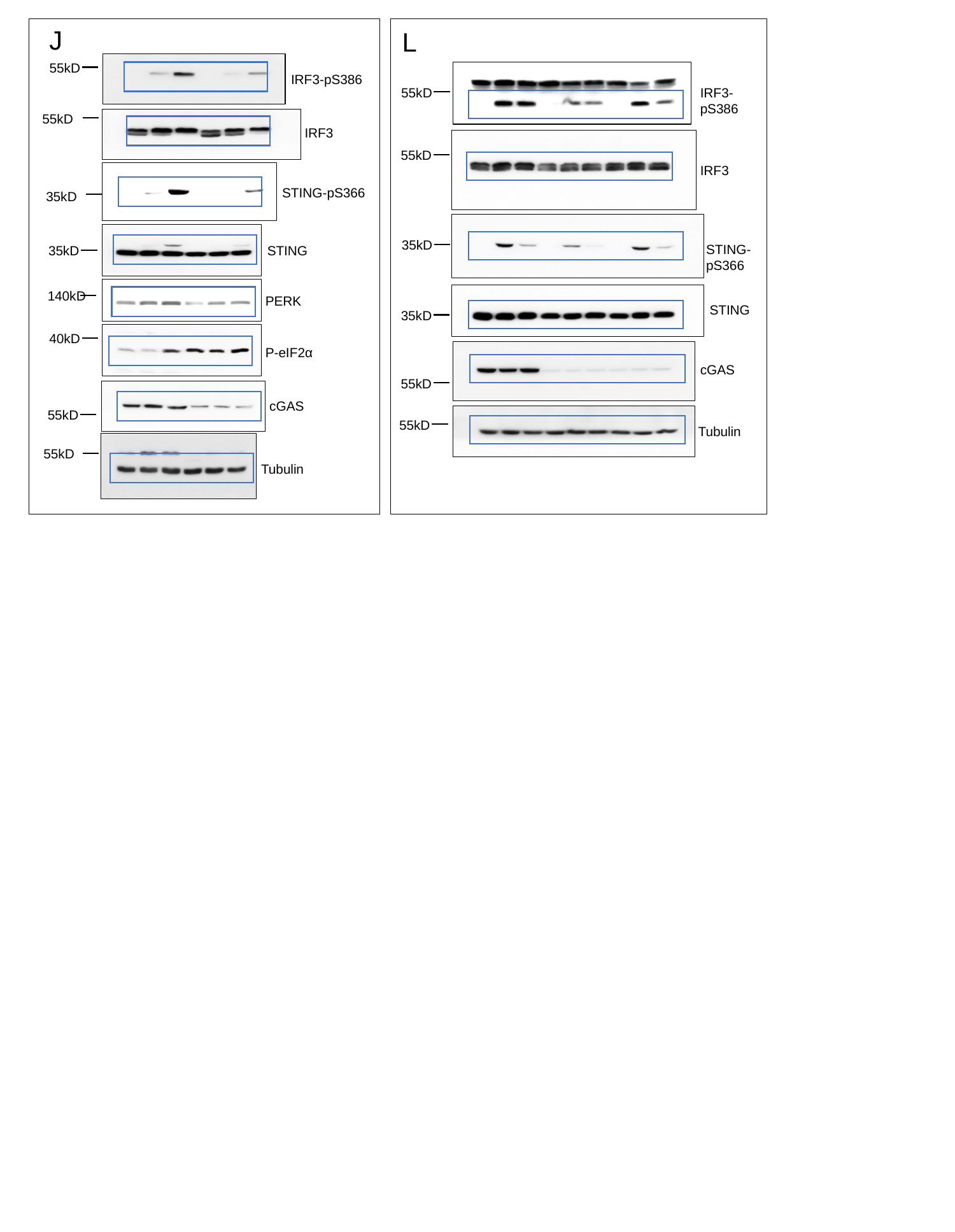

L
55kD
IRF3-pS386
55kD
IRF3
35kD
STING-pS366
STING
35kD
cGAS
55kD
55kD
Tubulin
J
55kD
IRF3-pS386
55kD
IRF3
STING-pS366
35kD
35kD
STING
140kD
PERK
40kD
P-eIF2α
cGAS
55kD
55kD
Tubulin
